# Gα_o1_ and Gα_o1_/Gα_o2_ deletion differentially affect hippocampal mossy fiber tract anatomy and neuronal morphogenesis

**DOI:** 10.1111/jnc.16248

**Published:** 2024-10-28

**Authors:** Markus Höltje, Anton Wolkowicz, Irene Brunk, Jens Baron, Gudrun Ahnert‐Hilger

**Affiliations:** ^1^ Institut für Integrative Neuroanatomie, Charité–Universitätsmedizin Berlin Corporate Member of Freie Universität Berlin and Humboldt‐Universität Zu Berlin Berlin Germany; ^2^ Laboratory of Neurobiology Max‐Planck‐Institute for Biophysical Chemistry and University of Göttingen Göttinge Germany

**Keywords:** Gα_o1_, Gα_o2_, hippocampal mossy fiber tract, neuronal development

## Abstract

The heterotrimeric G‐protein αo subunit is ubiquitously expressed in the CNS as two splice variants Gα_o1_ and Gα_o2_, regulating various brain functions. Here, we investigated the effect of single Gα_o1_, Gα_o2_, and double Gα_o1/2_ knockout on the postnatal development of the murine mossy fiber tract, a central pathway of the hippocampal connectivity circuit. The size of the hippocampal synaptic termination fields covered by mossy fiber boutons together with various fiber length parameters of the tract was analyzed by immunohistochemical staining of the vesicular Zinc transporter 3 (ZnT3) or Synaptoporin at postnatal days 2, 4, 8, 12, 16, and in the adult. Ultimately, Gα_o1_ knockout resulted in a reduced developmental growth of synaptic mossy fiber terminal fields by 37% in the adult *Stratum lucidum* and by 30% in the total mossy fiber tract size. Other morphological parameters such as projection length of the infrapyramidal bundle of the tract were increased (+52% in Gα_o1_^−/−^ mice). In contrast, Gα_o2_ knockout had no effects on the mossy fiber tract. Moreover, by using primary heterozygous and homozygous Gα_o1_ knockout hippocampal cultures, we detected a strongly pronounced reduction in axon and dendrite length (−50% and −38%, respectively) as well as axon and dendrite arborization complexity (−75% and −72% branch nodes, respectively) in the homozygous knockout. Deletion of both splice variants Gα_o1_ and Gα_o2_ partially rescued the in vivo and completely reconstituted the in vitro effects, indicating an opposing functional relevance of the two Gα_o_ splice variants for neuronal development and synaptic connectivity.

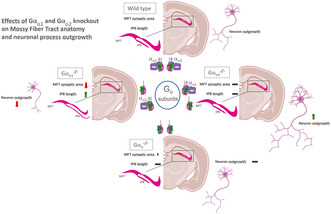

## Graphical abstract

Go proteins are among the most abundant proteins in the brain. Here we show that knockout of Ga_o1_ affects the development of the hippocampal mossy fiber tract (MFT), a pivotal part of the information flow through this brain area. Ga_o1_^−/−^ mice exhibit reduced synaptic termination fields of the MFT and an altered course of the infrapyramidal bundle (IPB) as part of the MFT. In line with this, cultured Ga_o1_‐depleted neurons show a reduced process outgrowth. Double knockout of Ga_o1_ and Ga_o2_ largely rescues these effects. Single knockout of Gα_o2_ does not affect MFT morphology besides being known to enhance process outgrowth of cultured neurons.

**Abbreviations**
CA: cornu ammonis
CNS: central nervous system
GPRIN1: G protein‐regulated inducer of neurite outgrowth 1
GTP: guanosine triphosphate
IPB: infrapyramidal bundle
Map2: microtubule‐associated protein 2
MFT: mossy fiber tract
NFP: neurofilament protein
SLU: stratum lucidum
SPB: suprapyramidal bundle
SyPo: synaptoporin
VGLUT1: vesicular glutamate transporter 1
ZnT3: zinc transporter 3

## 1 INTRODUCTION

Neuronal communication fundamentally relies on the action of G protein‐coupled transmembrane receptors that transduce their signals via activation of heterotrimeric G proteins thereby mediating binding of GTP to the Gα subunit and release of Gβγ subunits to interact with their effectors (Pierce et al., 2002). Among the so far 16 identified Gα subunits (Wettschureck & Offermanns, 2005), Gα_o_ is one of the most abundant proteins in the brain (Solis & Katanaev, 2017; Strittmatter et al., 1991), and failure of Gα_o_ signaling is crucially involved in pathophysiological conditions like altered cAMP signaling in movement disorders (Muntean et al., 2021). A plethora of recent studies report on genetic variants of the *GNAO1* gene in humans (from which both known splice variants Gα_o1_ and Gα_o2_ are generated) leading to severe early‐onset epileptic encephalopathy. Moreover, developmental delay, mild adolescent/adult‐onset dystonia in movement disorders like Parkinsonism, as well as intellectual disability occur (Decraene et al., 2024; Knight et al., 2023; Solis et al., 2024; Wirth et al., 2022). However, it is not clear how the two splice variants of Goα contribute to these pathologies.

So far, it was described that Gα_o1_ deletion markedly reduced body growth and life span and impaired motor function, while its sole presence (specific deletion of Gα_o2_) promoted axonal growth of cultured neurons (Baron et al., 2018). The clinical picture of *GNAO1* gene variants and the opposing effects of Gα_o1_ and Gα_o2_ in the dopaminergic system we had observed in the past (Baron et al., 2018) prompted us to gain more insight into the role of the GNAO1 splice variant Gα_o1_ in neuronal morphogenesis and development. Therefore, we morphometrically compared the course of the hippocampal mossy fiber tract development between wild‐type, Gα_o1_, Gα_o2_, and Gα_o1/2_ knockout mice. These studies were complemented by in vitro morphometrical analysis of cultured neurons obtained from Gα_o1_ or Gα_o1/2_ deletion mutants.

One of the characteristic features of the hippocampus represents its largely unidirectional and trisynaptic projection path involving the dentate gyrus and the pyramidal cells of the CA3 and the CA1 regions (Andersen et al., 1969; Hjorth‐Simonsen, 1973). The connection of the dentate granule cells with CA3 pyramidal cells occurs via the mossy fiber tract, whose axons run through the hilus of the dentate gyrus thereby giving rise to collaterals terminating at interneurons (Acsády et al., 1998) and terminate in the *Stratum lucidum* above the CA3 region, named for its homogeneous transparency in unstained tissue. Mossy fiber axons run along a main fiber tract (suprapyramidal bundle) but can also reach the CA3 region via the infrapyramidal bundle. Infrapyramidal fibers cross the pyramidal cell layer and penetrate into the deep layers in the *Stratum lucidum*, thus converging with the suprapyramidal tract (Blaabjerg & Zimmer, 2007; Claiborne et al., 1986). Communication with their postsynaptic target cells is achieved via three different synaptic terminals: These include the characteristic large mossy fiber terminals, filopodia from the mossy fiber terminals, and smaller en passant synapses at varicosities (Claiborne et al., 1986; Frotscher et al., 1991). From the CA3 pyramidal cells, the excitation chain spreads via glutamatergic Schaffer collaterals toward CA1 pyramidal cells. Because of the largely unidirectional information flow through the hippocampus, the mossy fiber tract is crucial for proper hippocampus function and disturbances within this fiber bundle have been described in association with brain pathologies such as epilepsy and schizophrenia (Abulaiti et al., 2022; Schmeiser et al., 2017). The trajectory of its path can easily be visualized by identifying its synaptic termination fields using immunostaining against either the vesicular Zinc transporter 3 (ZnT3) or Synaptoporin, which are both heavily enriched in the mossy fiber terminals (Barth et al., 2011; Hitt et al., 2012; Imig et al., 2020; Wiqas et al., 2020).

Using immunohistochemistry on murine brain sections, we studied the effects of genetic Gα_o1_, Gα_o2_, and Gα_o1/2_ deletion on the anatomy of the mossy fiber tract starting from postnatal day 2 to adulthood. In addition, effects on neuron morphology in Gα_o1_ and Gα_o1/2_ knockout strains were analyzed in cultured hippocampal neurons.

## 2 MATERIALS AND METHODS

### 2.1 Animals

Gα_o1_^−/−^ (NIE/Birnb Go1alpha 129SV/C57BJ/6), Gα_o2_^−/−^ (NIE/Birnb Go2alpha 129SV/C57BJ/6), and Gα_o1/2_^−/−^ (NIE/Birnb Goalpha 129SV/C57BJ/6) splice variant‐specific mutant mice were obtained from the laboratory of Lutz Birnbaumer, bred in the local animal facility and genotyped as described (Dhingra et al., 2002; Jiang et al., 1998). Mutant and WT littermates were obtained by interbreeding of heterozygous parents. Institutional approval (approval number T0119/11) was obtained for sacrificing the animals used in this study. Adult animals were kept with 3–4 cage companions in a space of 360 cm^2^, enriched with a hiding place, nest‐building material, and ad libitum access to water and food. Pups were kept with their mother before sacrificing.

### 2.2 Antibodies

Synaptic connections of mossy fiber axon terminals were stained by polyclonal sera directed against either the vesicular Zinc transporter 3 (ZnT3, #197003) or Synaptoporin (cat. no.102002), both obtained from Synaptic Systems (Göttingen, Germany). For double stainings with ZnT3, a mouse monoclonal Synaptoporin antibody from Synaptic Systems was applied (cat. no.102011). A polyclonal antiserum against the vesicular glutamate transporter 1 (cat. no. 135303, VGLUT1), monoclonal anti‐Synaptobrevin and anti neurofilament H antibodies were also from Synaptic Systems (cat. no.104211 and cat. no. 171111). Morphology of cultured hippocampal neurons was visualized by a polyclonal antiserum against microtubule‐associated protein 2 (MAP2, cat. no. AB5622) and a monoclonal antibody against neurofilament protein of 200 kDa (cat. no. AB5256) both from Chemicon International (Hofheim, Germany). A monoclonal antibody against both splice variants of Gα_o_ (Gα_o1_ and Gα_o2_, clone 101.1) was previously described (Winter et al., 2005). A polyclonal antibody preferentially recognizing Gα_o1_ was from Santa Cruz (cat. no. 13532, Santa Cruz, CA, USA).

Secondary antibodies were Alexa 594 goat anti‐rabbit and Alexa 488 goat anti‐mouse (cat. no. A‐11037 and cat. no. A‐11029, Thermo Fisher, Waltham, MA, USA). Red fluorescence is displayed in magenta in all figures. For western blotting, peroxidase‐conjugated anti‐mouse and anti‐rabbit IgG were obtained from Vector Laboratories (cat. no. PI‐2000 and cat. no. PI‐1000; Newark, CA, USA).

### 2.3 Western blotting

For immunoblotting, whole brains from adult wild‐type, Gα_o1_^−/−^, and Gα_o1/2_^−/−^ mice were homogenized (tissue was processed in PBS using a glass‐Teflon homogenizer applying 10 strokes at 900 rpm with protease inhibitors added). Homogenates were spun down at 1500 *g* for 10 min, and the resulting supernatant devoid of cell nuclei was diluted in Laemmli buffer and submitted to SDS‐PAGE. To this end, samples were dissolved in Laemmli buffer, heated for 5 min at 95°C, and transferred to Nitrocellulose membranes by semi‐dry blotting using 10% SDS gels. The membranes containing the transferred proteins were blocked with 5% dry milk and 0.1% Tween‐20 in 20 mM Tris for 1 h at RT, followed by incubation with the respective primary antibodies overnight, secondary antibodies were applied for 2 h at room temperature. Washing between steps was carried out with 20 mM Tris buffer. Visualization was performed by Enhanced Chemiluminescence.

### 2.4 Brain sections and mossy fiber tract analysis

20 μm coronal cryosections from 3 to 4 animals of either sex per genotype and age (postnatal days P2, P4, P8, P12, P16 and 2–3 months of age, for Gα_o2_ knockout mice, only adult animals (2–3 months old) were looked at) were analyzed. Adult animals were transcardially perfused with 4% paraformaldehyde. Prior to perfusion, mice were anesthetized by subcutaneous injection of 10–12 mg/100 g ketamine and 1–1.5 mg/100 g xylazine. Mice of ages P2‐P16 were killed by cervical dislocation without prior anesthesia, and brains were immersion‐fixed by 4% paraformaldehyde. In total, 84 animals were sacrificed for immunohistochemistry. Per brain, 10–14 sections from equivalent wild‐type/knockout rostral‐caudal locations were selected using section numbers and anatomical landmarks, including size and shape of the hippocampus and ventricles. Mossy fiber tract projections were visualized by staining against ZnT3 (P12 to adulthood) or Synaptoporin (Sypo, all ages). The following substructures of the mossy fiber tract were morphometrically investigated from images taken by a 10× lens and analyzing ZnT3 or SyPo stainings (see also Figure 2b): length of the infrapyramidal bundle (IPB), segmented‐line drawn through the middle of the IPB from the end of the pyramidal cell layer to the most distal point below the pyramidal cell layer; length of the suprapyramidal bundle (SPB), segmented‐line drawn through the middle of the SPB (axon bundle superior to the pyramidal cell layer) from the division of the hilar projections into the suprapyramidal/infrapyramidal bundles to the SPB/Stratum lucidum (SLU) junction (defined as the point a line originating from the tip of the SLU and perpendicular to a line extending from the vertex of the SLU hits the tract, see also Figure 2b); length of the SLU, segmented‐line drawn through the middle of the SLU from the SPB/SLU junction to the tip of the SLU; total mossy fiber tract length, SLU + SPB length + a segmented‐line drawn from the origin of the SBP to the origin of the tract within the hilus of the dentate gyrus. In addition, the area of the SLU and the rest of the mossy fiber tract was determined by the stainings. For control, the medio‐lateral (width) as well as the ventro‐dorsal (height) dimensions of the hippocampus formation were determined. Morphometrical measurements were done by using the analysis tools of Leica application software (Version 2.8.1, Leica Microsystems CMS GmbH, Balgach, Switzerland).

### 2.5 Hippocampal cell culture and morphometrical measurements

Neurons were prepared from fetal brains of wild‐type, heterozygous, or homozygous Gα_o1_ or Gα_o1/2_ knockout mice at embryonic day 16 (E16). Following the killing of pregnant mice by cervical dislocation, embryos were dissected from the mother and killed by decapitation. Per experiment (each was repeated three times) and genotype, 6 animals were sacrificed. In total, 108 animals at E16 and 18 adult mothers were used for neuron cultures. Pieces of the whole hippocampus formation including all CA regions and dentate gyrus were rinsed with PBS, then with dissociation medium (modified Eagle medium (MEM) supplemented with 10% fetal calf serum, 100 IU/L insulin, 0.5 mM glutamine, 100 U/mL penicillin/streptomycin, 44 mM glucose, and 10 mM HEPES buffer) followed by mechanical dissociation. Following centrifugation, neurons were resuspended in starter medium (serum‐free neurobasal media supplemented with B27, 0.5 mM glutamine, 100 U/mL penicillin/streptomycin, and 25 μM glutamate) and plated at a density of 2 × 10^4^ cells/well on poly‐l‐lysine/collagen precoated glass coverslips. All ingredients were obtained from Gibco/BRL Life Technologies (Eggenstein, Germany).

Morphometrical measurements were carried out after 5 days in vitro.

Morphometric analysis of cultured neurons was performed by a Leica DMLB microscope using an ×40 objective lens. Total length, overall number of branching nodes of axons and dendrites (axon and dendrites were discerned by morphology and NFP/Map2 double stainings), 2D Sholl analysis, and determination of branch order occurrence were analyzed morphometrically using the Neurolucida software (MicroBrightField, Williston, VT, USA). The parameter “axon length” represents the integral total length of all visible parts of an axon, including its higher‐order branches (Ahnert‐Hilger et al., 2004). Experiments were carried out using three independent culture preparations. Typically, six coverslips were analyzed per preparation and genotype, and 5 neurons randomly taken were evaluated on each coverslip. Data from 90 individual neurons were pooled and given as means ± SEM.

### 2.6 Statistical analyses

Assessment of the normality of data was verified by the Shapiro–Wilk test for all data prior to parametric testing (Graph Pad Prism 8.0). All parameters looked at p values were greater than the chosen alpha level of 0.05. Statistical significance for knockout effects on mossy fiber tract morphology was determined by a two‐tailed, unpaired Student's *t*‐test on the basis of a significance level of *p* < 0.05 (MS Excel 2019). Genotype‐related differences in cultured neurons were statistically analyzed using the Kruskal–Wallis and Mann–Whitney U test for individual experiments, and t‐tests for the sum analysis on the basis of a significance level of *p* < 0.05 (Excel 2019). No test for outliers was conducted for in vivo and in vitro data. For predetermination of sample size, experiences from former publications of our group dealing with the effects of Go proteins were used (Baron et al., 2013, 2018; Brunk et al., 2008). In bar chart graphs, mean values from pooled animals or cultures are shown.

## 3 RESULTS

### 3.1 Characterization of Gα_o1_ knockout mice

**FIGURE 1:**
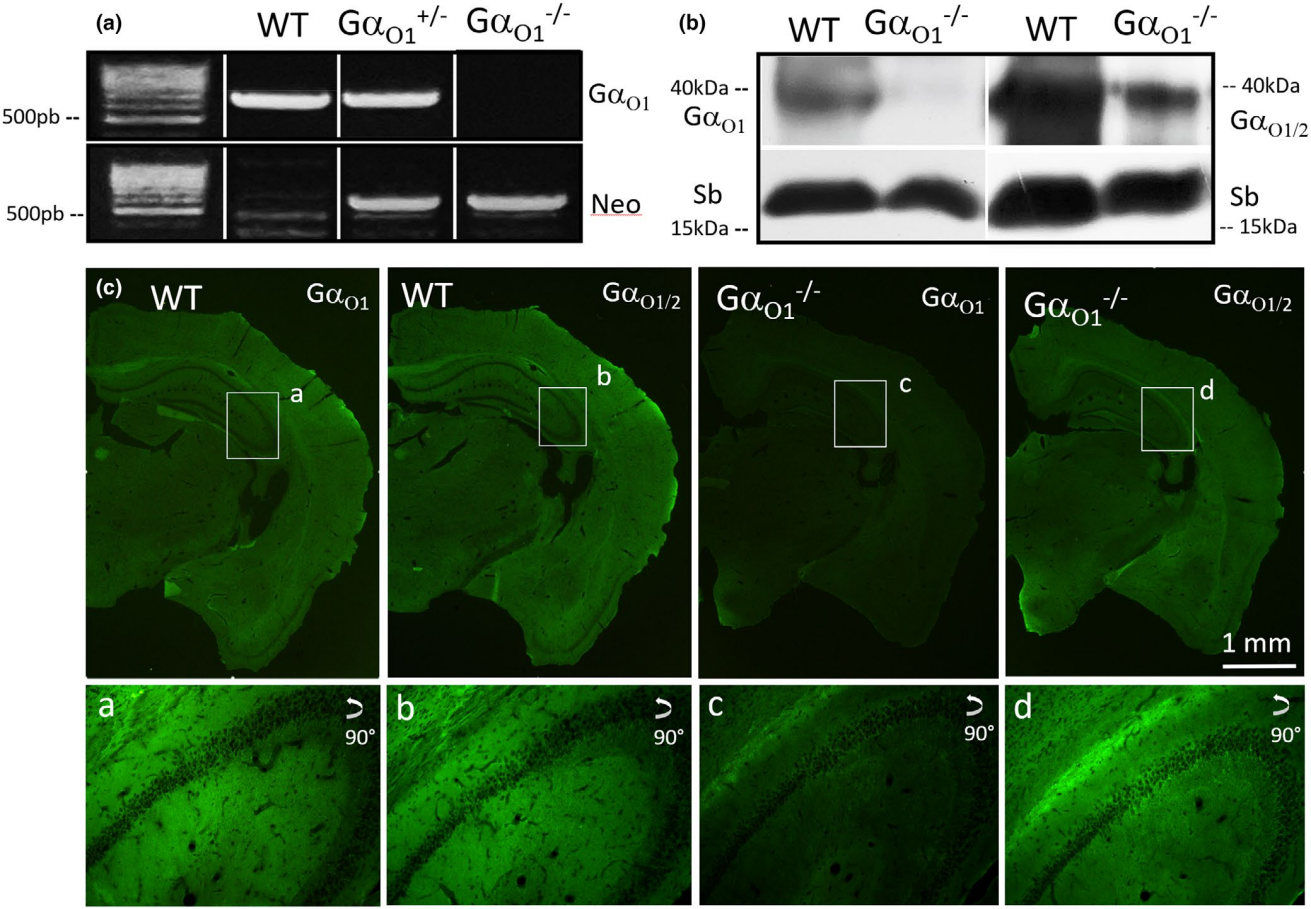
Knockout of Gα_o1_ as verified by genotyping, western blot, and immunohistochemistry. (a) Exemplary genotyping of wild‐type and Gα_o1_ heterozygous and homozygous knockout animals. Genomic template DNA was isolated from ear punches and amplified by PCR. In the wild‐type and heterozygous mice, Gα_o1_ DNA was clearly detectable but absent in homozygous mice. Neomycin (Neo) cassette DNA confirmed knockout. (b) Western blot analysis of Gα_o1_ and Gα_o2_ protein expression. Brain homogenates of wild‐type and Gα_o1_^−/−^ mice were stained by an antibody preferentially recognizing Gα_o1_ and an antibody detecting both Gα_o1_ and Gα_o2_. Vesicular Synaptobrevin (Syb) was used as control protein. Both Gα_o_ antibodies detected a major band below 40 kDa in the wild‐type that was largely absent in the knockout when using the antibody preferentially detecting Gα_o1_ and still detected by the Gα_o1/2_ antibody. (c) Immunohistochemical analysis of Gα_o1_ and Gα_o2_ expression in wild‐type and Gα_o1_^−/−^ mice. Coronal brain sections of adult mice of either strain were stained with the same antibodies as shown above. In the wild‐type, both antibodies showed a ubiquitous immune reaction in virtually all brain areas. Again, immunostaining against Gα_o1_ was largely absent in the knockout, while Gα_o2_ was still clearly detectable by the Gα_o1/2_ antibody (see insets for hippocampal CA3 area).

To investigate the effects of Gα_o_ subunit deletion on the morphology of the mossy fiber tract, we initially characterized Gα_o1_ knockout mice with regard to genotype and phenotypical parameters. Pups were obtained as littermates from heterozygous parents and genotyping was performed for all animals used for analysis to confirm correct knockout (Figure 1a). In addition, deletion of Gα_o1_ was confirmed on protein level by biochemical and immunohistochemical methods. For western blotting, we used two antibodies already characterized in earlier studies that show either a strong preference for Gα_o1_ or recognize both splice variants Gα_o1_ and Gα_o2_ (Brunk et al., 2008). Using brain homogenates from adult wild‐type and Gα_o1_^−/−^ mice, specific deletion of Gα_o1_ and remaining presence of Gα_o2_ was demonstrated by the two antibodies (Figure 1b). Using the same antibodies, knockout of Gα_o1_ was confirmed by immunofluorescence on brain sections. The Gα_o_ protein is one of the most abundant proteins in the brain, accordingly, both antibodies exhibited a positive immune signal in virtually all brain areas in the wild‐type (Figure 1c). Staining by the Gα_o1_ antibody was strongly reduced (remaining immune signal representing residual reactivity to Gα_o2_), while presence of Gα_o2_ was affirmed by the Gα_o1/2_ detecting antibody in the knockout mice. Phenotypically, homozygous deletion of Gα_o1_ results in marked changes such as strong developmental retardation in body size and weight, slightly reduced brain weight, and a life span shortened to 22.3 days (Figure 1a–c). Heterozygous deletion had a much less pronounced effect on the parameters looked at.

### 3.2 Changes in hippocampal mossy fiber tract anatomy caused by the deletion of Gα_o1_

**FIGURE 2:**
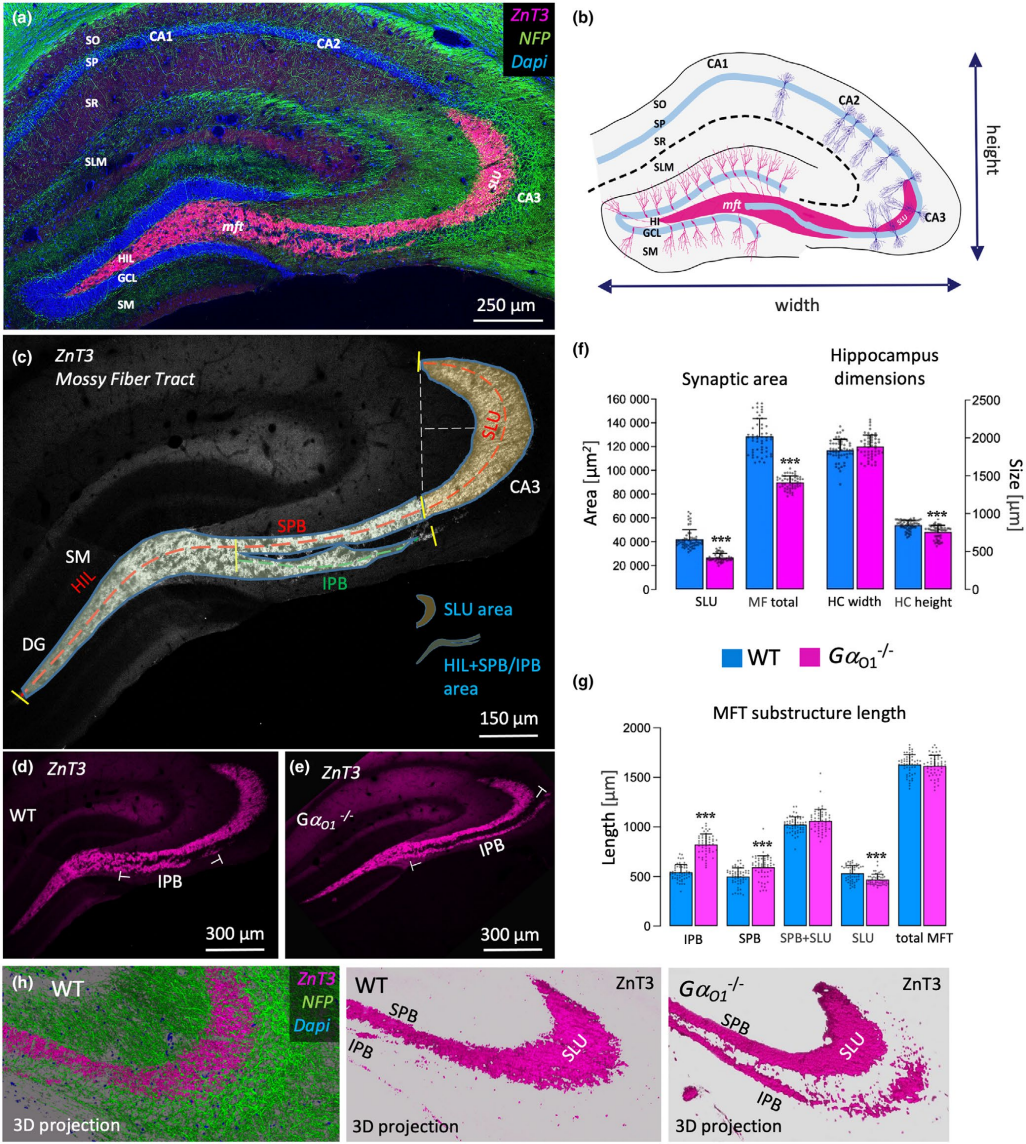
Altered projections and reduced size of mossy fiber synaptic areas in the hippocampus of Gα_o1_^−/−^ mice. (a) Coronal section from an adult wild‐type mouse brain was immunostained for the vesicular Zinc transporter 3 (ZnT3, heavily marking the mossy fiber tract (mft), and the axonal marker neurofilament heavy chain (NFP)), together with Dapi to depict the gross anatomy of the hippocampus and its layered organization. The mossy fiber tract originates from the granule cells of the dentate gyrus, passes through the hilus, and terminates at the *Stratum lucidum* (SLU) forming synapses with the CA3 pyramidal cells. (b) Schematic representation of the hippocampus formation and the mossy fiber tract bundles. Before reaching the *Stratum lucidum*, the mossy fiber tract splits up into two bundles running above and below the pyramidal cells. Arrows indicate the two distance lines measured to determine the gross size of the hippocampus formation in the medio‐lateral (width) and dorso‐ventral (height) axis. GCL, granule cell layer; HIL, *hilum*; SLM, *Stratum lacunosum/moleculare*; SM, Stratum moleculare; SO, *Stratum oriens*; SP, *Stratum pyramidale*; SR, *Stratum radiatum*; (c) Representative section of a wild‐type hippocampus stained for ZnT3 to demonstrate the mossy fiber tract substructures analyzed for quantification. Mossy fibers project from the hilus above and below the pyramidal cell layer, termed suprapyramidal and infrapyramidal bundle (SPB and IPB) before terminating in the *Stratum lucidum* (SLU). Length of the IPB, SPB, SPB + SLU, SLU alone as well as the overall length of the tract from the tip of the hilus to the tip of the SLU were determined (see yellow lines for portions of the tract). Additionally, the area of the SLU and the rest of the mossy fiber tract were quantified. (d, e) Representative images of adult wild‐type (same as in c) and a Gα_o1_^−/−^ hippocampi stained for ZnT3 are shown. Most strikingly, the MFT appears thinner and the IPB appeared to be longer in the knockout animals than in the wild‐type. (f, g) Quantification of measurements. Both the SLU and overall synaptic mossy fiber area were markedly reduced in Gα_o1_^−/−^ animals. General hippocampus width was not significantly altered, hippocampus height was slightly reduced in KO animals. Most prominently, the length of the IPB was reduced in wild‐type compared to the knockout animals, accompanied by a slight reduction in the SBP length. The length of the SLU was moderately reduced in G*α*_*O1*_ knockout animals. The other parameters were unaltered. Bars show means ± SD from *N* = 4 pooled animals each WT and Gα_o1_^−/−^, 51 (knockout) and 54 (wild‐type) mossy fiber tracts per genotype ***p ≤ 0.001 (h) Confocal imaging and 3D reconstruction from z‐stacks showing ZnT3 expression together with surrounding axons stained by NFP at a wild‐type SLU area (left panel). 3D reconstruction of ZnT3 staining at the SLU area showing the differences in SLU size and IPB length between wild‐type (middle panel) and Gα_o1_^−/−^ mice (right panel).

Following the initial characterization of Gα_o1_ knockout mice, we aimed to investigate the effects on the developmental morphology of the anatomically well‐defined and functionally in‐depth characterized mossy fiber tract (MFT). The MFT represents a bundle of unmyelinated axons that pass the information coming from the dentate gyrus to the CA3 hippocampal area, therefore representing a crucial element of information flow through the hippocampus. Its large synaptic terminals can be visualized by established markers such as the vesicular Zinc transporter (ZnT3) or Synaptoporin that allow for a very accurate determination of its course. Because of the observed minor phenotypical changes elicited by heterozygous knockout of Gα_o1_ (that also showed the same effect direction as the full knockout), we decided to focus on homozygous knockout animals. Staining of adult wild‐type mice hippocampi with ZnT3 resulted in the expected staining pattern of the MFT, originating from the hilus of the dentate gyrus and projecting toward the *Stratum lucidum* (SLU) area of the CA3 region (Figure 2a,b). Before reaching the SLU area, the bundle splits up into two parts, one running above the pyramidal cell layer (suprapyramidal bundle, SPB) and one running underneath it (infrapyramidal bundle, IPB). Typically, the IPB projects into the pyramidal cell layer before reaching the SLU (Figure 2c). By evaluating ZnT3 stainings, we morphometrically determined various length parameters of the MFT such as IBP and SPB, combined length of the SBP and SLU, length of the total MFT from the tip of the hilum to the tip of the SLU, as well as the length of the SLU alone. In addition, the size of synaptic termination areas of the SLU and the total MFT were measured. To minimize unspecific morphological effects of the knockout on MFT anatomy caused by general alterations in hippocampal size, we also determined the gross size of the hippocampi analyzed. When comparing adult brains of wild‐type and Gα_o1_^−/−^ mice, it became evident that knockout mice exhibited longer IPBs that projected far beyond the SBP/SLU transition zone usually observed as the entrance of the IBP into the pyramidal cell layer in the wild‐type (Figure 2d,e). Morphometrical analysis detected more alterations in MFT anatomy in the knockout (Figure 2f,g): deletion of Gα_o1_ resulted in a profound reduction in the size of both the SLU area alone and the total MFT area (SLU ‐37%, total MFT −30%). On the other hand, the IBP length was increased by 52% (see also Figure 2h), together with more moderate changes in SBP length (+19%) and SLU length (−12%). Hippocampus dimensions were only affected by a moderate reduction of height (−11%) in the knockout. Taken together, the most prominent alterations in Gα_o1_^−/−^ mice compared to the wild‐type emerged as reductions in the size of the synaptic termination areas of the MFT and a longer infrapyramidal bundle.

**FIGURE 3:**
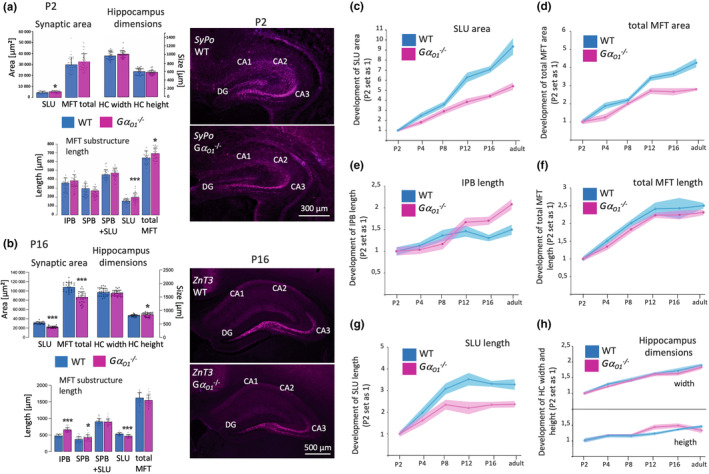
Development of differences in mossy fiber tract anatomy between wild‐type and Gα_o1_^−/−^ mice. Measurements of mossy fiber tract parameters of wild‐type and *Gα*_*o1*_^*−/−*^ mice from postnatal day 2 into adulthood. Analysis was based on either ZnT3 (P12, P16) or Synaptoporin (P2‐P8) stainings. (a) At P2, only very slight differences in the size of the synaptic area of the SLU and the total length of the mossy fiber tract were observed. All other parameters analyzed remained unchanged. (b) At P16, changes of analyzed parameters in *Gα*_*o1*_^*−/−*^ mice were most pronounced for reduction in synaptic areas and increased length of IBP. Bars show means ± SD from *N* = 3 pooled animals each WT and G*α*_o1_^−/−^ per age, 30 mossy fiber tracts per genotype and age. *p ≤ 0.05; ***p ≤ 0.001 (c–h) Absolute changes displayed in (a) and (b) and Figure S3 for the individual time points are summarized as relative changes from P2 to adulthood. Values for P2 (both for wild‐type and *Gα*_*o1*_^*−/−*^ mice) were set as 1. (c) Fold changes in SLU area. (d) Fold changes in total MFT area. (e) Fold changes in IPB length. (f) Fold changes in total MFT length. (g) Fold changes in SLU length. (h) Fold changes in hippocampus dimensions. Data show means ± SD, *N* values correspond to the information given in a and b.

To elucidate the developmental effects of Gα_o1_ deletion on MFT anatomy, we investigated the same size parameters in postnatal stages P2, P4, P8, P12, and P16. Analysis revealed that expression of ZnT3 emerges at P12, earlier developmental stages showed no signal (Figure S2a). Synaptoporin, on the other hand, was already detectable at P2 and persisted into adulthood (Figure S2b, see S2c for comparison with VGLUT expression). Both mossy fiber tract markers showed identical staining patterns of the MFT and could be used from P12 and older as equivalent tools to recognize the MFT anatomy (Figure S2d). Overall, effects of Gα_o1_ knockout in earlier stages P2, P4, and P8 resulted in moderate alterations of individual parameters that mostly increased with age as in the total MFT area (Figure 3a,b; Figure S3 for P4‐P12, from P4 on a persistent reduction in the size of total MFT area was observed, with −21% at P16). This was accompanied by a reduction in the size of the SLU area (−16% at P4, −7% at P8, −30% at P12, −28% at P16) and SLU length (constant reduction from P12 on). Differences in IPB length (as observed in the adult) with respect to shorter length in the wild‐type became significant from P12 on (+21% in Gα_o1_^−/−^ mice, +38% at P16). Conversely, width and height of the hippocampus formation did not show major variations that could account for the differences in MFT anatomy between the two genotypes at the investigated time points (Figure 3a,b; Figure S3).

When summarizing the effects of Gα_o1_ deletion on MFT anatomy as relative growth (earliest inspected age P2 was set as 1 for both wild‐type and knockout, absolute parameters were mostly identical between strains at this age), it became obvious that the largest differences in developmental growth were in the SLU area size (Figure 3c). Adult wild‐type animals had increased the SLU area around 9,3fold from P2, while the growth was only 5,4fold in Gα_o1_^−/−^ mice. Total MFT area had increased 4,2fold in the wild‐type and 2,7fold in the knockout (Figure 3d). Interestingly, growth of IPB length was similar between strains till P8 with slightly lower values in the knockout before growth stagnated in the wild‐type from P12 on and continued in Gα_o1_^−/−^ mice (length in the knockout more than doubled from P2 to the adult, wild‐type IBP showed an almost 1,5fold increase). Total MFT length (Figure 3e) showed similar growth values for both genotypes, growth stagnated around P12 between 2,3 and 2,4fold (Figure 3f), SLU growth also stopped at that age with a 3,3fold increase from P2 to the adult in wild‐type and a 2,4fold increase in the knockout (Figure 3g). Hippocampal width and height exhibited comparable growth processes between the strains during postnatal development (Figure 3h). Generally, the size of the synaptic areas, especially in the wild‐type, increased with age to a greater extent than the length of the MFT substructures.

### 3.3 Double knockout of Gα_o1_ and Gα_o2_ very moderately affects mossy fiber tract development

**FIGURE 4:**
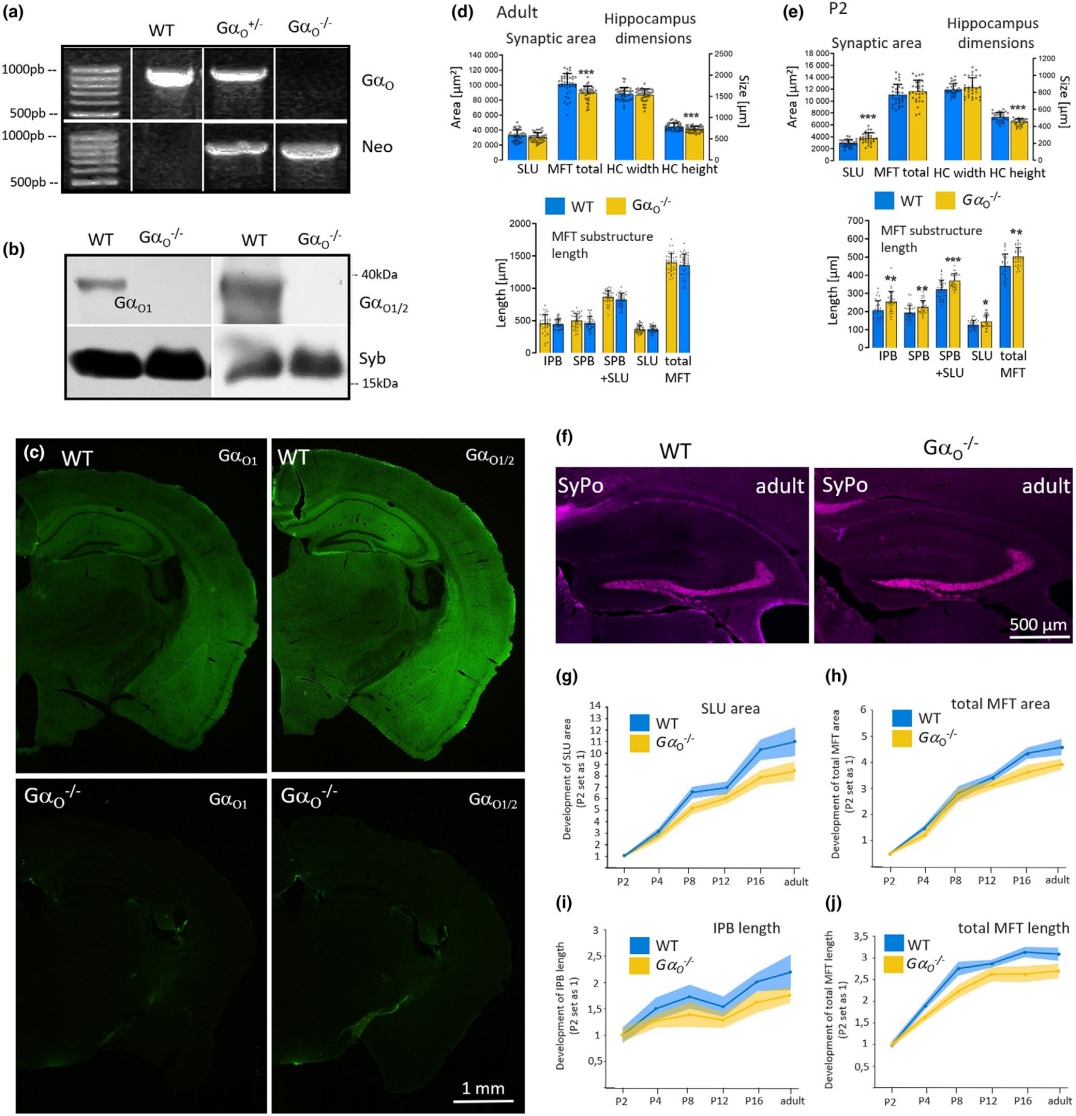
Double knockout of Gα_o1_ and Gα_o2_ largely restores inhibitory effects of single Gα_o1_ deletion on synaptic area and alterations in mossy fiber tract substructure length. (a) Exemplary genotyping of wild‐type and Gα_o_ (Gα_o1_ and Gα_o2_) heterozygous and homozygous knockout animals. Genomic template DNA was isolated from ear punches and amplified by PCR. In the wild‐type and heterozygous mice, Gα_o_ DNA was clearly detectable but absent in homozygous mice. Presence of Neomycin (Neo) cassette DNA confirms knockout in heterozygous and homozygous animals. (b) Western blot analysis of Gα_o1_ and Gα_o2_ protein expression. Brain homogenates of wild‐type and Gα_o_^−/−^ mice were stained by an antibody preferentially recognizing Gα_o1_ and an antibody detecting both Gα_o1_ and Gα_o2_. Vesicular Synaptobrevin (Syb) was used as internal control protein. Both Gα_o_ antibodies detected a major band below 40 kDa in the wild‐type that was fully absent in the knockout when using either antibody. (c) Immunohistochemical analysis of Gα_o1_ and Gα_o2_ expression in wildtype and Gα_o_^−/−^ mice. Coronal brain sections of adult mice of either strain were stained with the same antibodies as shown above. In the wild‐type, both Gα_o1_ and Gα_o1/2_ antibodies showed a ubiquitous signal in virtually all brain areas. Again, immunostaining against Gα_o1_ and Gα_o2_ was negative for both antibodies in the knockout. (d, e) Quantification of mossy fiber tract measurements of adult and P2 wild‐type and Gα_o_^−/−^ mice based on Synaptoporin stainings. In the adult, total MFT synaptic area and, very moderately, hippocampus height were reduced in knockout animals. All other parameters remained unchanged in the knockout. At P2, moderate changes appeared in the synaptic SLU area and all MFT length parameters with slightly higher values in the knock‐out. Bars show means ± SD from *N* = 4/3 pooled animals each WT and Gα_o_^−/−^ adult/P2, 40/30 mossy fiber tracts per adult/P2 genotype *p ≤ 0.05; **p ≤ 0.01; ***p ≤ 0.001 (f) Representative coronal sections of adult wild‐type and Gα_o_^−/−^ hippocampi show no obvious changes in mossy fiber tract anatomy as judged by Synaptoporin stainings. (g–j) Absolute changes are displayed as relative changes from P2 to adulthood. Values for P2 (both for wild‐type and Gα_o_^−/−^ mice) were set as 1. (g) Fold changes in SLU area. (h) Fold changes in total MFT area. (i) Fold changes in IPB length. (j) Fold changes in total MFT length. Data show means ± SD, *N* values correspond to the information given in (d, e).

In Gα_o1_ knockout animals, Gα_o2_ is still—compared to the wild‐type at even elevated levels—expressed (Baron et al., 2018). We therefore investigated the outcome of Gα_o1_/Gα_o2_ depletion on the development of the mossy fiber tract. Genotyping again assured correct homozygous or heterozygous deletion of the *GNAO1* gene from which both splice variants are generated (Figure 4a). Western blotting from adult brain homogenates confirmed the complete absence of Gα_o_ proteins by the two antibodies detecting preferentially Gα_o1_ or both splice variants (Figure 4b). Depletion of Gα_o_ was confirmed by immunofluorescence on brain sections. Both antibodies exhibited a positive immune signal in virtually all brain areas in the wild‐type but showed no immune signal in the knockout (Figure 4c). Phenotypical changes in Gα_o1_/Gα_o2_ double knockout (Gα_o_) mice were looked at and effects on body, and brain weight were comparable to Gα_o1_ knockout animals. However, Gα_o_^−/−^ mice showed a slightly increased median life span of 32.1 days (compared to 22.2 days in Gα_o1_^−/−^ mice; Figure S1).

In accordance with the studies in Gα_o1_ knockout animals, the developmental time course of Gα_o_ deletion effects on the MFT anatomy was investigated. In adult mice, the total MFT area was only moderately reduced by 11.8% in the knockout, while the SLU area was not significantly altered between genotypes, strongly contrasting the situation in Gα_o1_^−/−^ brains (Figure 4d). Hippocampus height was reduced by 7.8%. All adult MFT substructure parameters were not affected by the knockout.

**FIGURE 5:**
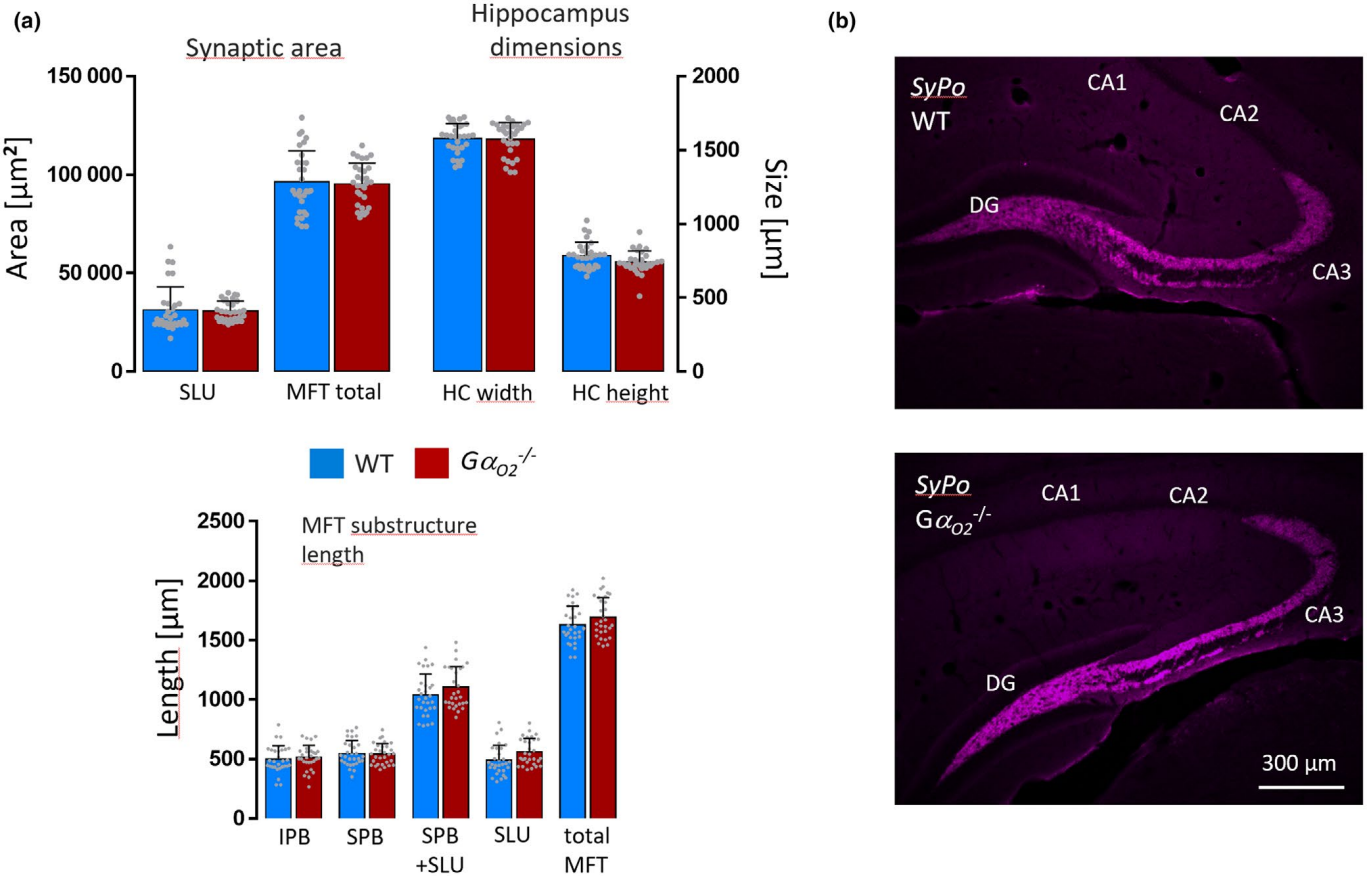
Mossy fiber tract anatomy in adult wild‐type and G*α*_o2_^−/−^ mice. Measurements of mossy fiber tract parameters of adult wild‐type and Gα_o2_^−/−^. Analysis was based on Synaptoporin stainings. (a) No differences in the size of the MFT synaptic area or Hippocampus dimensions were observed. All other MFT parameters analyzed also remained unchanged. (b) Representative images of adult wild‐type and Gα_o2_^−/−^coronal brain sections stained for Synaptoporin. Bars show means ± SD from *N* = 3 pooled animals each WT and Gα_o2_^−/−^per age, 30 mossy fiber tracts per genotype and age.

At the start of the observation period, the SLU area had already grown 20% larger than in the wild‐type at P2 (Figure 4e). In line with this, all MFT length parameters were moderately increased in Gα_o_^−/−^ brains, only hippocampus height was slightly reduced by the deletion. The similar appearance of the mossy fiber tract anatomy in adult wild‐type and Gα_o_^−/−^ mice is illustrated, contrasting the situation in adult Gα_o1_^−/−^ mice (Figure 4f). During development from P4 to P16, synaptic area size and MFT length parameters as well as hippocampus dimensions showed no or only minor changes, e.g., at P4 and from P12 on a moderate significant reduction in total MFT synaptic area emerged (Figure S4). To compare and sum up the developmental growth in wild‐type and Gα_o_ knockout mice, the MFT anatomy is again displayed as relative growth. From P2 on (set as 1, most absolute values were slightly higher in the knockout at the start of the observation period) the MFT growth curve of Gα_o_^−/−^ mice was running moderately lower than in the wild‐type. For the SLU area (Figure 4g) values from P2‐adult were 8,4‐fold versus 11‐fold (knockout vs. wild‐type), for total MFT area (Figure 4h) 7,8fold versus 9,1‐fold, for growth of the IPB (Figure 4i) 1,7‐fold vs. 2,1‐fold (marking one of the most pronounced differences to the situation in Gα_o1_^−/−^ mice), and total MFT length growth values were 2,7‐fold vs. 3,5‐fold (Figure 4j). Length of SLU and hippocampus dimensions showed nearly identical fold changes between the genotypes (data not shown). Given the observed positive effects of single Gα_o2_ knockout on axonal outgrowth of cultured neurons (Baron et al., 2013), we also analyzed putative effects on MFT anatomy in adult Gα_o2_^−/−^ knockout animals. No differences between wild‐type and knockout animals, however, regarding length or synaptic MFT area parameters were detected, contrasting the in vitro situation (Figure 5).

### 3.4 Knockout of Gα_o1_ strongly reduces axonal and dendritic outgrowth in cultured neurons, rescued by double Gα_o1/2_ knockout

In addition to the immunohistochemical analysis of single Gα_o1_ and double Gα_o1/2_ knockout studies on the mossy fiber tract growth, we investigated axonal and dendritic outgrowth of cultured primary hippocampal neurons obtained from both Gα_o_ subunit knockdown strains. Morphometrical Neurolucida analysis measurements were based on neurofilament (NFP) and microtubule‐associated protein 2 (Map2) stainings used as axonal and dendritic markers, respectively.

**FIGURE 6:**
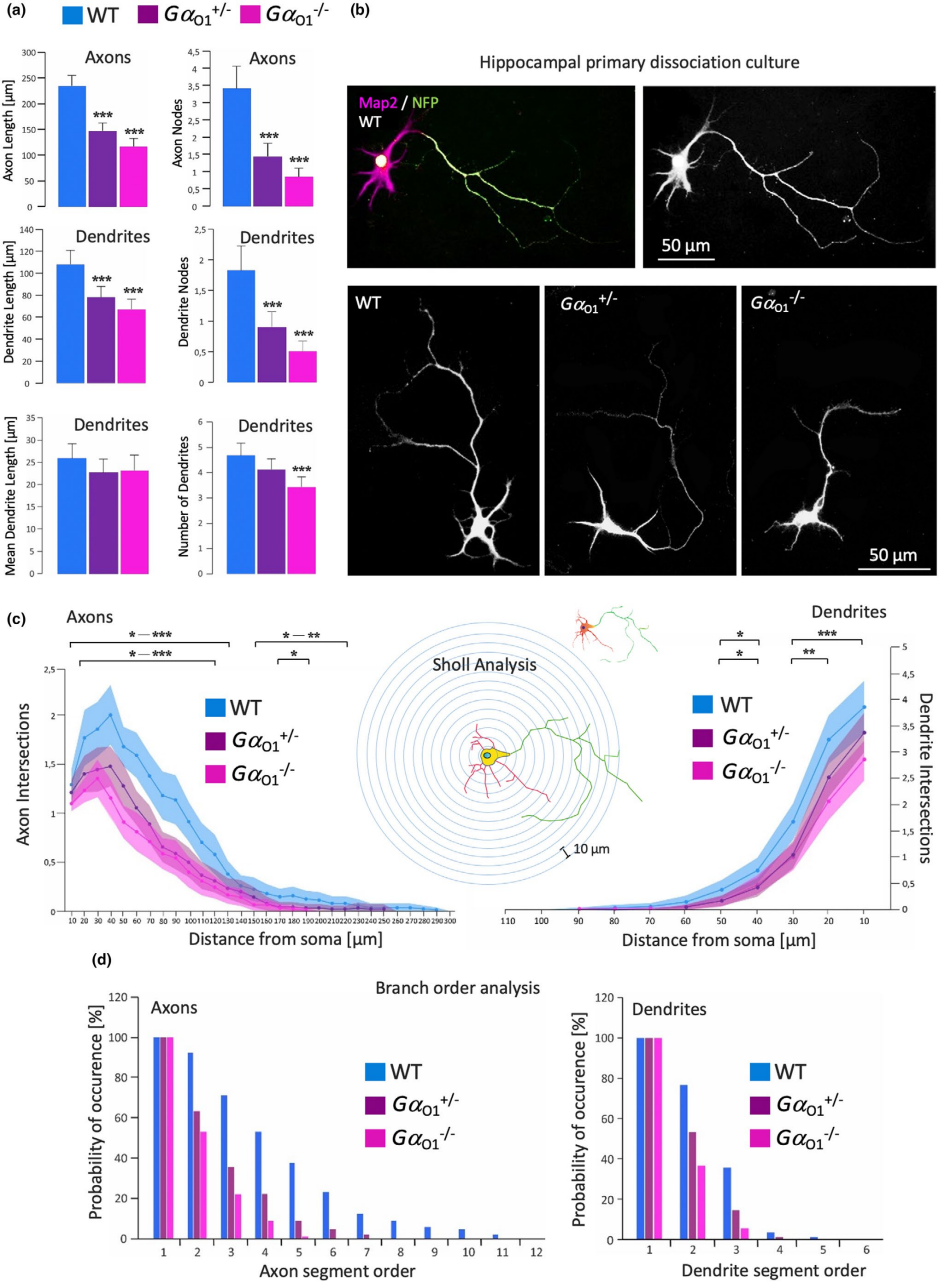
Knockout of Gα_o1_ reduces axonal and dendritic length and branching in cultured hippocampal neurons. Morphometrical measurements of cultured hippocampal neurons. (a) Hippocampal neurons were obtained from embryonic wild‐type, Gα_o1_^+/−^, and Gα_o1_^−/−^ brains and maintained for 5 days in culture. Following fixation, neurons were stained for Microtubule‐associated protein 2 (Map2) and neurofilament protein (NFP) as dendritic and axonal markers. Morphometrical parameters were analyzed using Neurolucida software. Both heterozygous and homozygous knockout resulted in significant reduction of axonal and dendritic length and branching, more pronounced in the homozygous genotype. In addition, Gα_o1_^−/−^ neurons showed a reduction in the number of dendrites developed. ***p ≤ 0.001 (b) Representative images of Map2 and NFP double stainings and alterations of neuronal morphology by the respective knockout types. (c) Sholl analysis of axonal (left) and dendritic (right) growth and complexity. A number of intersections of neuronal branches with circles centered on the soma with 10 μm increment in the distance from the soma were counted. Wild‐type neurons showed a significantly higher number of axon intersections over a broad range of distances from the soma and reached further distances especially than Gα_o1_^−/−^ neurons. Heterozygous neurons showed a less pronounced effect. Number of dendritic intersections was also higher in the wild‐type than in knockout neurons, the maximal distance reached was comparable for the three genotypes. Summarized p values indicated by asterisks in the upper rows apply to differences between wild‐type and Gα_o1_^−/−^ neurons, asterisks in the lower row indicate differences between wild‐type and Gα_o1_^+/−^ neurons. *p ≤ 0.05, **p ≤ 0.005, ***p ≤ 0.001 (d) Branch order analysis of axonal and dendritic growth. Compared to the wild‐type, both heterozygous and homozygous knockout neurons displayed a reduced development of higher‐order branches (expressed as probability of occurrence in % of neurons). Dendrites at DIV 5 generally show a less complex branching pattern than axons, but, however, also exhibit a reduced expression 2nd‐ to 4th‐order branches. Data in (a), (c) and (d) are given as means ± SEM from three independent experiments, 90 neurons were analyzed per individual condition.

First, Gα_o1_ knockdown neurons were quantified with the heterozygous genotype included in the analysis. Axonal length (−38% in Gα_o1_^+/−^ and − 50% in Gα_o1_^−/−^ neurons) and branch points (−59% axon nodes in Gα_o1_^+/−^ and −75% in Gα_o_^−/−^ neurons) were strongly reduced (Figure 6a,b). Similar robust effects were observed for dendritic length (−28% in Gα_o1_^+/−^ and − 38% in Gα_o1_^−/−^ neurons) and dendrite nodes (−50% in Gα_o1_^+/−^ and −72% in Gα_o1_^−/−^ neurons). Additionally, the number of dendrites developed was reduced by 28% in homozygous knockout neurons. Sholl analysis of homozygous Gα_o1_^−/−^ neurons showed a significantly reduced number of axon intersections over almost the full range of distances measured, heterozygous Gα_o1_^+/−^ neurons exhibited a less pronounced reduction in intersections. Qualitatively, the effects on dendrite intersections were comparable to the effects observed on axons for the two knockout strains (Figure 6c). In addition, we performed a branch order analysis to quantify the branch orders developed, with higher orders indicating a more complex branching pattern (Figure 6d). Wild‐type neurons grew branches up to the 11th order, while heterozygous Gα_o1_^+/−^ cells developed branches up to the 7th order and homozygous Gα_o1_^−/−^ neurons up to the 5th order. Dendrites at 5 days in vitro do not show an extensive branching pattern; however, a reduction in the development of 2nd, 3rd, and 4th order was observed for both knockout strains. These findings in Gα_o1_^−/−^ cultures contrast the observations in Gα_o2_^−/−^ neurons where both axonal growth and arborization were promoted in the sole presence of Gα_o1_ (Baron et al., 2013).

**FIGURE 7:**
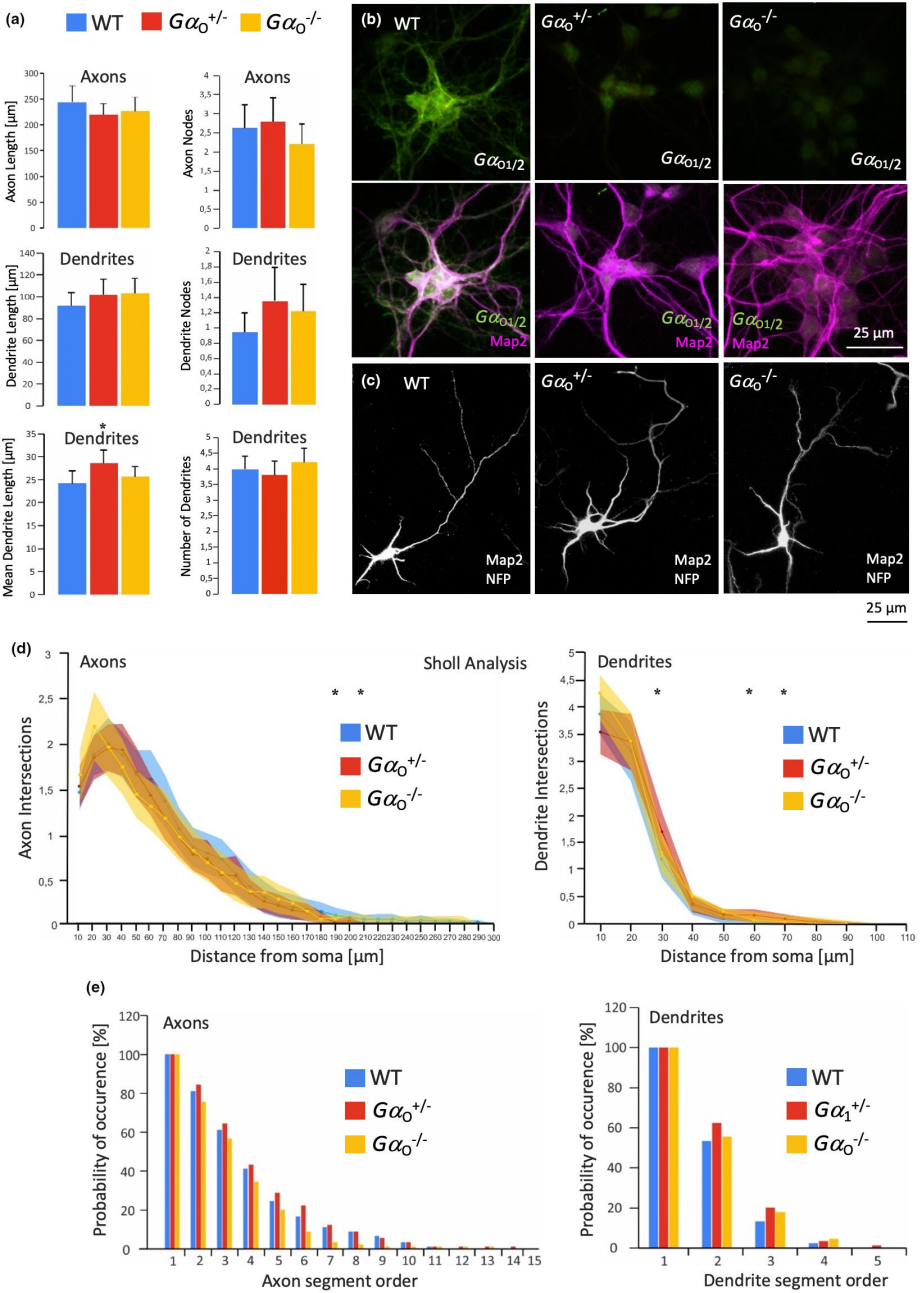
Double knockout of Gα_o1_ and Gα_o1_ restores wild‐type axonal and dendritic length and branching in cultured hippocampal neurons. Morphometrical measurements of cultured hippocampal neurons. (a) Hippocampal neurons were obtained from embryonic wild‐type, Gα_o_^+/−^ and Gα_o_^−/−^ brains and maintained for 5 days in culture. Following fixation, neurons were stained for Microtubule‐associated protein 2 (Map2) and neurofilament protein (NFP) as dendritic and axonal markers. Morphometrical parameters were analyzed using Neurolucida software. Mean dendritic length was slightly increased in Gα_o_ heterozygous mice, all other dendritic and axonal parameters looked at were unchanged. *p ≤ 0.05 (b) Representative images of Gα_o1/2_ and Map2 double staining in cultured neurons. Both knockout types result in a loss of immune signal. (c) Representative images of combined Map2 and NFP stainings to demonstrate the similar morphology of all three genotypes. (d) Sholl analysis of axonal (left) and dendritic (right) growth and complexity. A number of intersections of neuronal branches with circles centered on the soma with 10 μm increment in the distance from the soma were counted. No significant changes were observed between wild and Gα_o_^−/−^ type neurons, and some minor changes were detectable between wild‐type and Gα_o_^+/−^ neurons. Asterisks apply to differences between wild‐type and Gα_o1_^+/−^ neurons. *p ≤ 0.05 (e) Branch order analysis of axonal and dendritic growth. Compared to the wild‐type, both heterozygous and homozygous knockout neurons developed similar axonal and dendritic branch orders. Data in (a), (d) and (e) are given as means ± SEM from three independent experiments, 90 neurons were analyzed per individual condition.

Remarkably, morphometrical analysis of complete Gα_o_ knockdown neurons depleted of both splice variants revealed almost no significant effects on axonal and dendritic growth and arborization except for a mild positive knockdown effect on mean dendritic length in Gα_o_^+/−^ neurons in culture (Figure 7a). Loss of Gα_o_ in cultured knockout neurons was verified by immunofluorescence (Figure 7b). Similar morphology between wild‐type, Gα_o_^+/−^, and Gα_o_^−/−^ neurons is illustrated by the combined NFP/Map2 stainings (Figure 7c). In accordance with the findings on overall axonal and dendritic length and branching, Sholl analysis also revealed no gross differences in neuronal morphology between the three strains (Figure 7d). The same holds true for the branch order analysis, which showed very similar development of axonal and dendritic segment orders (Figure 7e).

Taken together, the current study demonstrates the effects of single Gα_o1_, Gα_o2_, and double Gα_o1/2_ knockout on the developmental morphology of the hippocampal mossy fiber tract, showing a reduced growth in the size of the synaptic mossy fiber termination fields in Gα_o1_^−/−^ mice associated mainly with an altered projection anatomy of the infrapyramidal bundle. Most effects were abolished or weakened by the double knockout, while single deletion of Gα_o2_ had no effect. Accompanying in vitro experiments showed a very accentuated reduction of axonal and dendritic growth by the knockout of Gα_o1_, while depletion of Gα_o2_ is known to promote process outgrowth. Knockout of both Gα_o1_ and Gα_o2_ nearly fully rescued these effects.

These novel findings, therefore, shed new light on the opposing effects two splice variants of the same protein can exercise on brain development and neuronal morphology.

## 4 DISCUSSION

In this study, we demonstrate that the single knockout of Gα_o1_ considerably reduced the size of the mossy fiber tract and its terminal synaptic fields with CA3 pyramidal neurons in the *Stratum lucidum* (SLU). When knocking out both splice variants, however, the observed effects of Gα_o1_ deletion were strongly reduced, or even abolished. Single knockout of Gα_o2_, on the other hand, had no effect on the mossy fiber tract.

Reduction of synaptic SLU and total MFT areas mainly developed from P12 on and later in Gα_o1_ knockout mice. Reductions rather resulted from a shrinking in MFT thickness than tract length since SLU length was only moderately shortened in Gα_o1_ knockout animals. Interestingly, overall hippocampus size was not affected, underlining the specificity of Gα_o1_ deletion effects. The decrease in MFT size likely resulted from a reduction in the number of synapses formed between granule cell mossy fiber axons and CA3 pyramidal cell dendrites in the SLU or interneurons in the hilar area.

The reduced formation of mossy fiber synapses could be because of a decreased axonal branching of granule cells axons thereby limiting the number of contacts formed with CA3 and/or hilar neurons. In line with this, we observed strong negative effects of Gα_o1_ knockout on axonal (and dendritic) outgrowth and branching in cultured hippocampal neurons. Although representing an unsorted culture system lacking identification of specific neuron subtypes, the reduced axonal branching complexity could also account for the observed in vivo effects.

Longer infrapyramidal bundles are another marked feature of the MFT in Gα_o1_^−/−^ mice. Strain‐specific variations in the distribution of murine hippocampal mossy fiber synapses with respect to the infrapyramidal bundle were observed earlier (Barber et al., 1974). Behavioral studies demonstrated that infra‐ and intrapyramidal mossy fiber projections are involved in the modulation of spatial orientation capabilities in the radial maze. Larger infra/intrapyramidal projections were associated with better learning capabilities in the spatial radial‐maze tasks (Crusio & Schwegler, 2005). Around the third postnatal week, the synaptic contacts of the mossy fibers on the so‐called “thorny excrescences” have matured (Amaral & Dent, 1981). During development, axons formed in abundance undergo elimination of unnecessary synaptic connections and regression in length with the infrapyramidal tract projecting longer towards and below the SLU than in the mature situation. Between P20 and P30, synapses are eliminated and axons partially regress in a semaphorin‐mediated fashion, so that around P30 the infrapyramidal portion of the mossy fiber tract is retracted away from the SLU (Bagri et al., 2003). In this respect, the observed longer IPB in the knockout might represent an unrefined developmental situation. So far it is unclear how Gα_o1_ signaling fosters synapse formation and triggers retraction of infrapyramidal fibers during development. The observed effects on MFT morphology could also lie in an altered persistent and activity‐dependent neurogenesis of granule cells in rodents, given that adult neurogenesis occurs and mainly affects the IPB genetically independently regulated from the SPB (Krebs et al., 2011; Römer et al., 2011). So far, no data are available on the Gα_o_‐dependent regulation of neurogenesis in the developing or adult dentate gyrus.

Gα_o_ occurs in two splice variants Gα_o1_ and Gα_o2_ (Hsu et al., 1990). So far, effects of Gα_o1_ depletion have been described in different neuronal systems including for the light response of retinal ON bipolar cells to photoreceptor input (Dhingra et al., 2002; Okawa et al., 2010). The development of the MFT appears to be mainly dependent on Gα_o1_ as seen by the changes in the KO animals. In contrast, Gα_o1/2_ depletion partially rescued the effects seen in the Gα_o1_ knockout while Gα_o2_ has no effects on the morphology of MFT. So, while the knockout of Gα_o2_ is sufficient to foster axonal outgrowth in culture neurons, it fails to produce in vivo effects on MFT anatomy, at least when knocked out in the presence of Gα_o1_. Given these data, it appears that depletion of Gα_o2_ is able to counterbalance Gα_o1_ knock‐out‐mediated effects on MFT development, but fails to further enhance MFT development above wild‐type levels in the presence of Gα_o1_. In this respect, Gα_o1_ seems to be more fundamentally involved in neuronal morphogenesis, since its knockout produced robust effects both in vitro and in vivo.

Irrespective of the splice variants, Gα_o_ expression and signaling are known to take place at growth cones and involve a multitude of effectors to regulate axon outgrowth and therefore demonstrate an involvement of Gα_o_ subunits in neuronal morphogenesis (Bromberg et al., 2008; Sharma et al., 2015). In this context, G protein‐regulated inducer of neurite outgrowth 1 (GPRIN1) was identified to colocalize with Gαo at the growth cone of neuronal cells and to promote neurite extension as a downstream target for Gαo in a Cdc42‐dependent fashion (Nakata & Kozasa, 2005). Early splice variant‐specific studies investigating the NGF‐induced neurite outgrowth in neuroendocrine PC‐12 cells showed an increased expression of Gα_o1_ accompanied by neurite extension (Andreopoulos et al., 1995). Up to now, further data on selective Gα_o1_ effects on neuronal morpho‐ and synaptogenesis are lacking. It is tempting to investigate how Gα_o1_ and/or Gα_o2_ signaling affect neuronal growth regulating cues such as phosphatase and tensin homolog (PTEN) which drives expansion of *Stratum lucidum* CA3 mossy fiber synaptic fields by persistent “mTOR” activation when selectively knocked out in dentate gyrus granule cells (Yonan & Steward, 2023).

Previously we showed that Gα_o1_ but not Gα_o2_ knockout mice exhibited motor deficits compared to their wild‐type littermates when examined by rota‐rod behavioral experiments because of an imbalance in the dopaminergic system (Baron et al., 2018). Deletion of Gα_o1_ alone is accompanied by a two‐ to threefold increase of Gα_o2_. In this respect, the observed effects on MFT morphology in Gα_o1_ knockout mice may also be attributed to an increased expression of Gα_o2_. A differential interaction of both splice isoforms with effectors of cytoskeletal dynamics like the Rap1 effector Rap1GAP involved in neurite outgrowth (Jordan et al., 2005) was proposed in a way that Gα_o2_ activity reduces Rap1 activity while Gα_o1_ might promote degradation of Rap1GAP and therefore increases Rap1 activity and enhances axonal growth in hippocampal neurons (Baron et al., 2013). Therefore, Gα_o1_ and Gα_o2_ can be considered as opposing regulators of different neuronal systems, since the respective knockout resulted in the described reduction (Gα_o1_, this study) or enhancement (Gα_o2_, Baron et al., 2013) of neuronal process length and arborization.

A large amount of clinically oriented literature reports on the pathophysiological effects of GNAO1 (the common gene for both Gα_o1_ and Gα_o2_) mutations leading to so‐called GNAO1 encephalopathies including symptoms such as epilepsy, movement disorders, developmental delay, or intellectual disability (Ludlam et al., 2024). So far, it remains unclear whether the different genetic loci affected result in complete or splice variant‐specific depletion of Gα_o_ function or a disrupted balance between Gα_o1_ and Gα_o2_. Mutations may also result in modified Gα_o1_ and/or Gα_o2_ proteins with altered functions compared to non‐mutated forms. This again may then lead to an imbalance between Gα_o1_ and Gα_o2_ function. Moreover, depending on local expression differences between the two splice variants, the impact on pathologies may vary. The fact that both Gα_o1_ and double knockout mice die early at medium ages of 3–4 weeks (even if a low proportion can survive for months) together with the observed general growth retardation in both strains clearly demonstrates that other organ systems than the CNS are fundamentally affected. Go proteins are not restricted to the CNS, but are also found in the heart or the endocrine system (Jiang & Bajpayee, 2009). The exact causes of death have not yet been determined in detail, but it is likely that the rescue of Gα_o1_ knockout effects by additional depletion of Gα_o2_ as demonstrated in this study is not equally effective in other systems. This might especially be the case in tissues or organs that per se express low levels of Gα_o2_. Generally, the knockout of Gα_o1_ results in a much more severe phenotype than the knockout of Gα_o2_, since the latter shows a normal life span with only moderate phenotypical alterations.

To sum up, to our knowledge, our study for the first time demonstrates Gα_o1_‐specific effects on the development of a fundamentally important CNS pathway, together with general morphogenic effects of Gα_o1_ depletion on neuron morphology that can be largely reversed by additional Gα_o2_ knockout.

## AUTHOR CONTRIBUTIONS

**Markus Höltje:** Conceptualization; investigation; writing – original draft; validation; visualization; supervision. **Anton Wolkowicz:** Investigation; validation; visualization; writing – review and editing; software. **Irene Brunk:** Investigation; writing – review and editing. **Jens Baron:** Investigation; writing – review and editing. **Gudrun Ahnert‐Hilger:** Conceptualization; funding acquisition; writing – review and editing; supervision.

## FUNDING INFORMATION

This work was supported by grants of the Deutsche Forschungsgemeinschaft (DFG) to GAH (GAH 67/10–1).

### PEER REVIEW

The peer review history for this article is available at https://www.webofscience.com/api/gateway/wos/peer‐review/10.1111/jnc.16248.

## Supporting information


Data S1.

Figure S1.

Figure S2.

Figure S3.

Figure S4.

Data S2.

Data S3.

## Data Availability

The data that support the findings of this study are available from the corresponding author upon reasonable request.
