# Supplementary figures and images for: Gαo1 and Gαo1/Gαo2 deletion differentially affect hippocampal mossy fiber tract anatomy and neuronal morphogenesis
Source: J Neurochem. 2024 Oct 28;169(2):e16248. doi: 10.1111/jnc.16248 (PMC11808459; doi:10.1111/jnc.16248)

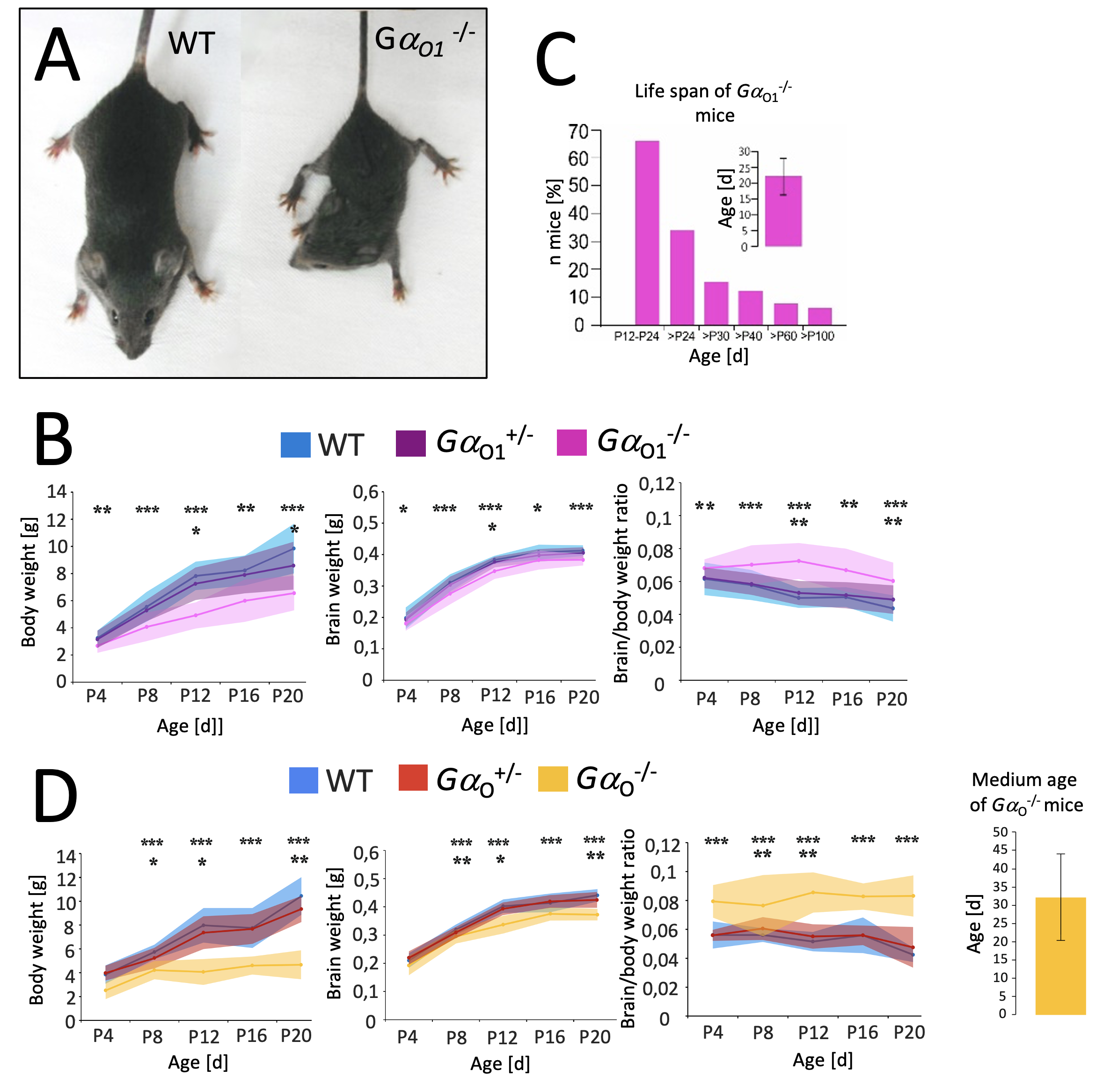

Supplement: Supplementary file 2 — Figure S1. [file JNC-169-0-s004.tif]

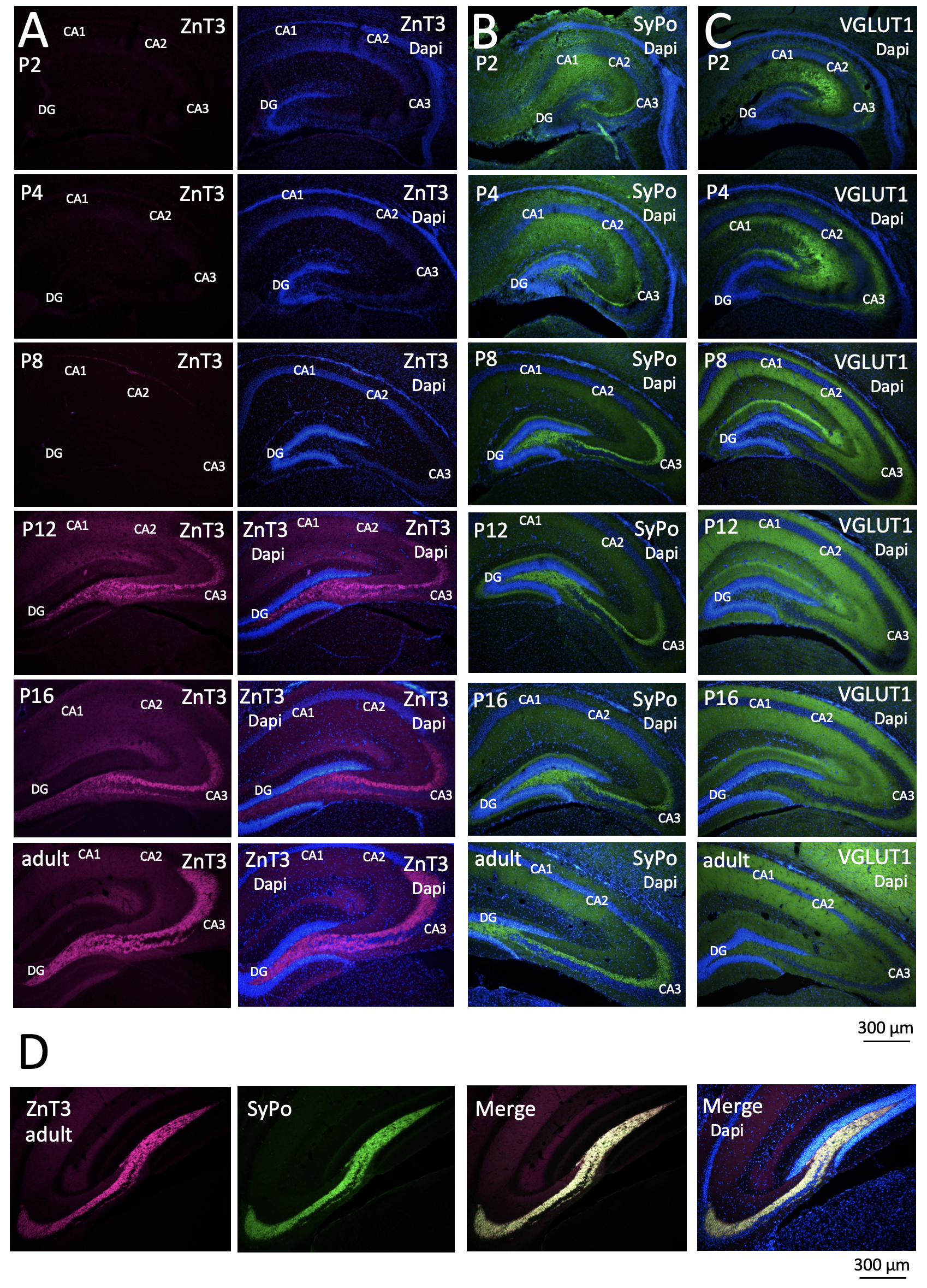

Supplement: Supplementary file 3 — Figure S2. [file JNC-169-0-s005.tif]

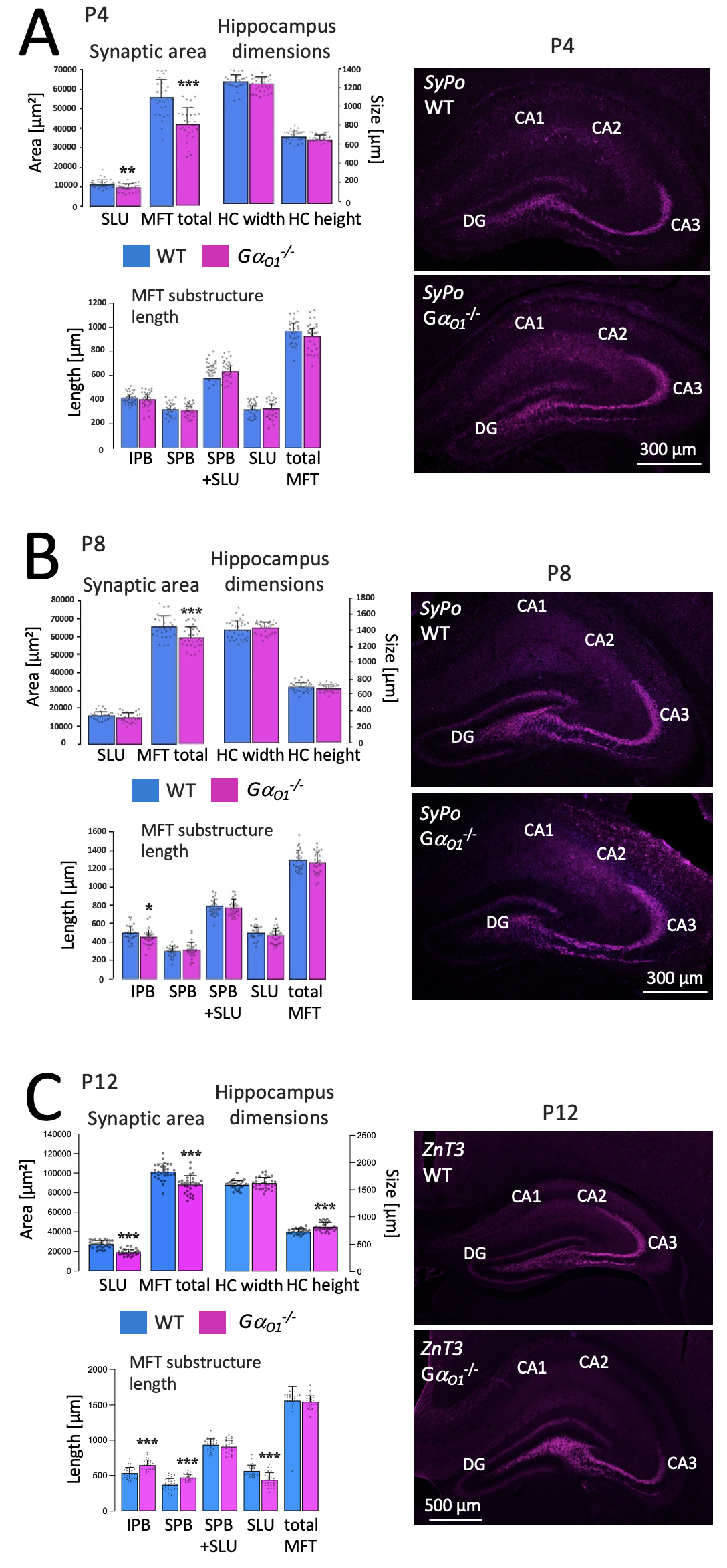

Supplement: Supplementary file 4 — Figure S3. [file JNC-169-0-s001.tif]

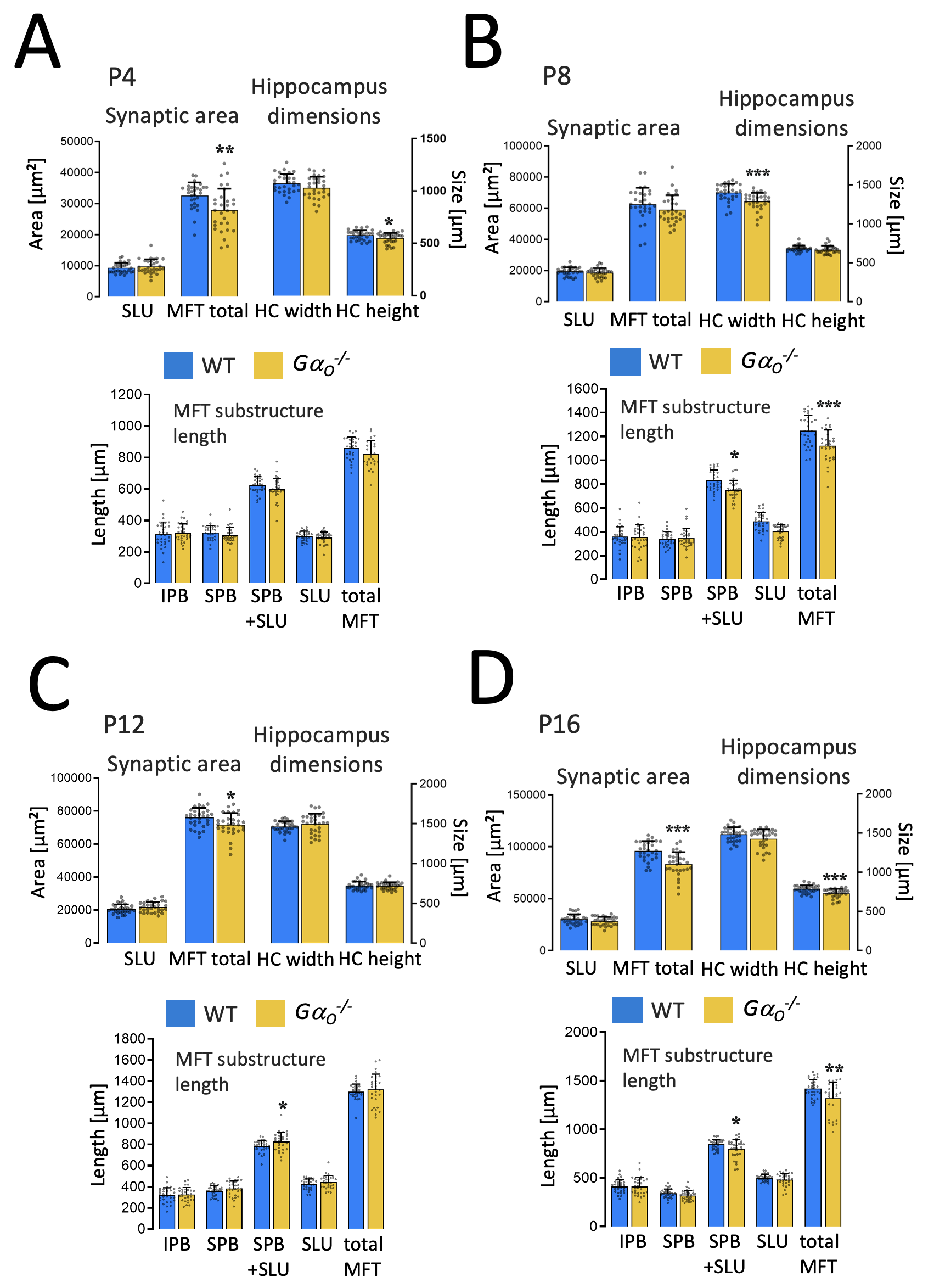

Supplement: Supplementary file 5 — Figure S4. [file JNC-169-0-s006.tif]
