# Supplementary material for: Gαo1 and Gαo1/Gαo2 deletion differentially affect hippocampal mossy fiber tract anatomy and neuronal morphogenesis
Source: J Neurochem. 2024 Oct 28;169(2):e16248. doi: 10.1111/jnc.16248 (PMC11808459; doi:10.1111/jnc.16248)
Supplement: Supplementary file 6 — Data S2. [file JNC-169-0-s003.docx]

**Gα_o1_** **and Gα_o1_** **/ Gα_o2_** **deletion differentially affect hippocampal mossy fiber tract anatomy and neuronal morphogenesis**

# Markus Höltje^1^*, Anton Wolkowicz^1^, Irene Brunk^1^, Jens Baron^1^, and Gudrun Ahnert-Hilger^2^

^1^ Institut für Integrative Neuroanatomie, Charité-Universitätsmedizin Berlin, corporate member of Freie Universität Berlin and Humboldt-Universität zu Berlin

^2^ Laboratory of Neurobiology, Max-Planck-Institute for Biophysical Chemistry and University of Göttingen, Germany

Supplemental data


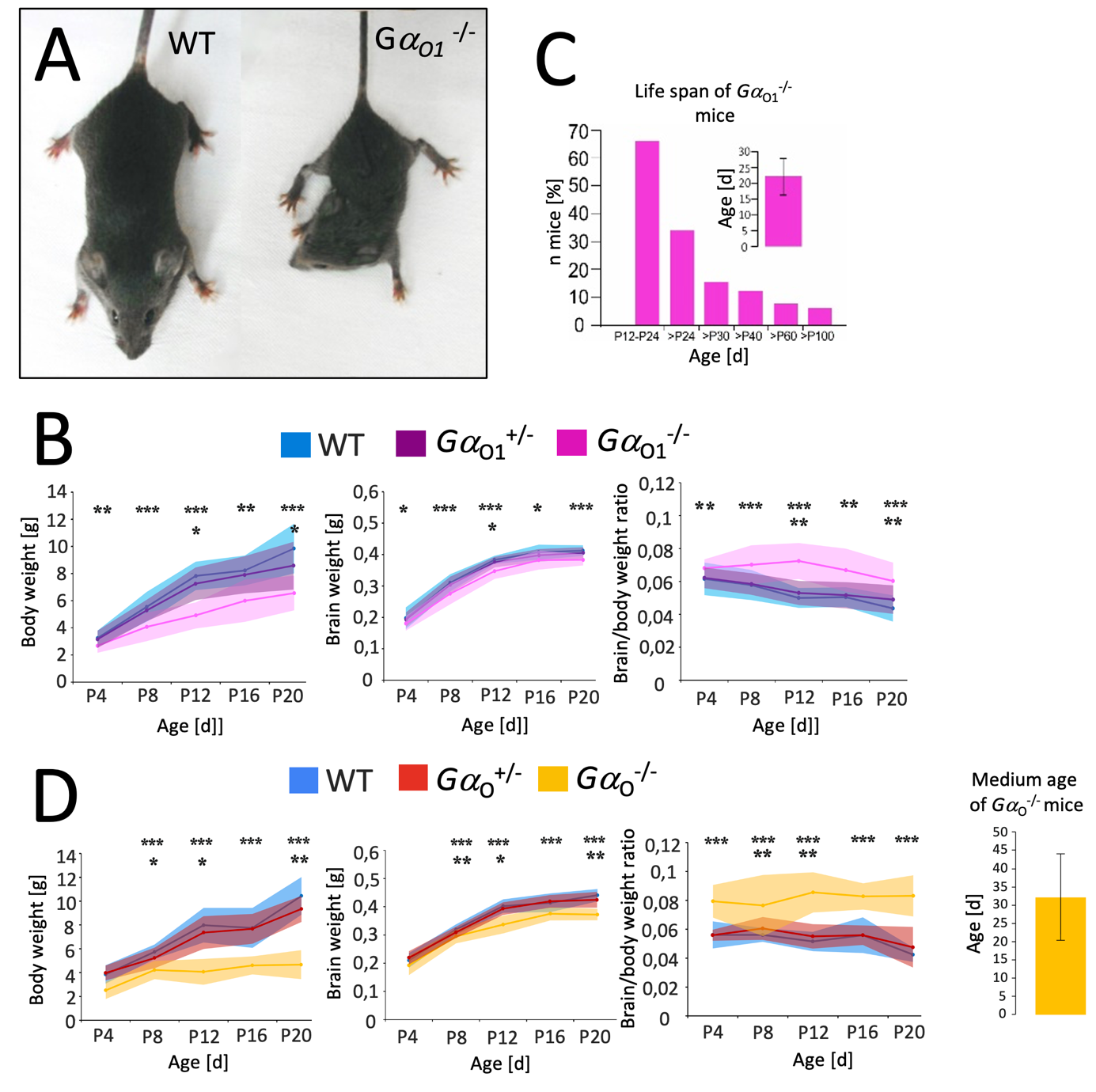


**Supplemental Figure 1**

**Single Gα_o1_ and double Gα_o1_ / Gα_o2_ knockout result in growth retardation and reduced live span**

A) Phenotypical appearance of adult wild type and Gα_o1_^-/-^ mice. Homozygous Gα_o1_ knockout mice exhibit a marked retardation in body size. B) Time course of postnatal body weight development in wild type, Gα_o1_^+/-^ and Gα_o1_^-/-^ mice (left panel). Determination of body weight was performed at postnatal (P) days P4, P8, P16 and P20. At all time points analyzed, homozygous mice were significantly lighter than wild type animals. Heterozygous mice showed significant alterations in body weight at P12 and P20. Corresponding postnatal development of brain weight in wild type, Gα_o1_^+/-^ and Gα_o1_^-/-^ mice (middle panel). At all time points analyzed, brains of homozygous mice were moderately lighter than the ones of wild type and heterozygous animals. Heterozygous mice showed no significant alterations in brain weigh compared to the wild type except for P12. Brain / body weight rations during postnatal development (right panel). The strong reduction in body size of homozygous mice compared to the relatively small reduction in brain size is reflected by the given ratios. C) Survival rates (given in %) of Gα_o1_^-/-^ mice at indicated postnatal time periods. Less than 10% of the animals survive to ages older than 100 days. Median age was calculated to 22,2 days. Data in B) are given as means ± SD (SD shown as transparent areas); N= 16-55 animals per condition of either sex. *P≤0,05; **P≤0,01; ***P≤0,001 upper asterisks wt vs. homoz KO., lower asterisks wt vs. heteroz KO C) N=37 animals D) Time course of postnatal body weight development (left panel) in wild type, Gα_o_^+/-^ and Gα_o_^-/-^ mice. Determination of body weight was performed at postnatal days P4, P8, P12, P16 and P20. From P8 on, homozygous mice were significantly lighter than wild type and heterozygous animals. Heterozygous mice showed only slight alterations in body weight at individual time points. Corresponding postnatal development of brain weight (middle panel). Also from P8 on, brains of homozygous mice were moderately lighter than the ones of wild type and heterozygous animals. Heterozygous mice showed very slight reductions in brain weigh compared to the wild type at individual time points. Brain/ body weight rations during postnatal development (right panel). The strong reduction in body size of homozygous mice compared to the relatively small reduction in brain size is reflected by the given ratios. Data are given as means ± SD (SD shown as transparent areas); N= 14-123 animals per condition of either sex. Median age was calculated to 32,1 days. *P≤0,05; ***P≤0,01; ***P≤0,001* upper asterisks wt vs. homoz KO., lower asterisks wt vs. heteroz KO


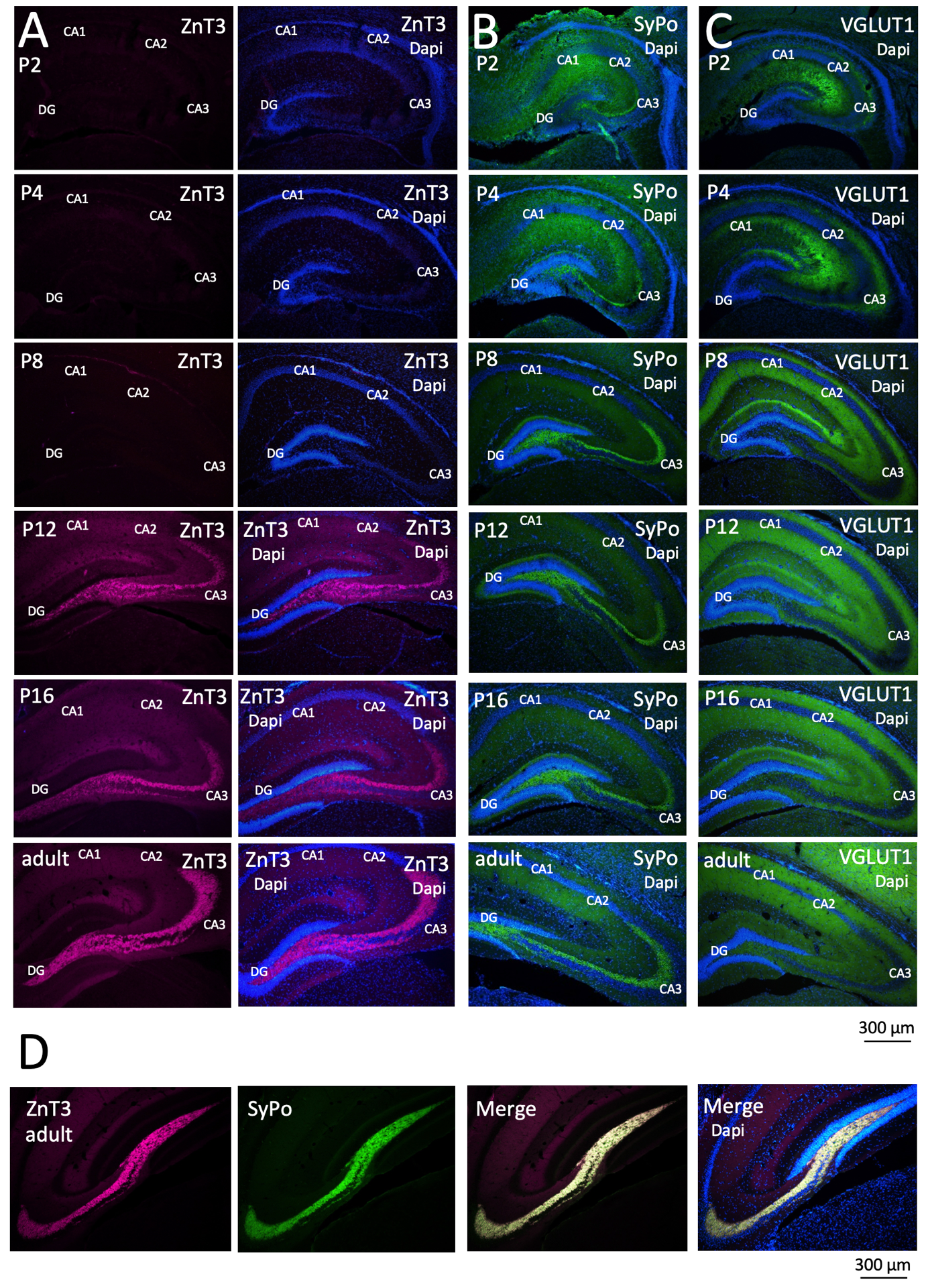


**Supplemental Figure 2**

**Postnatal development of mossy fiber tract marker proteins and the general excitatory synaptic marker VGLUT1**

Coronal sections from postnatal days 2, 4, 8, 12, 16 and adult wild-type mouse brains were immunostained for either ZnT3 (A), Synaptoporin (SyPo, B), or vesicular glutamate transporter 1 (VGLUT1, C). Immunohistochemical analysis shows that expression of ZnT3 is largely restricted to the mossy fiber tract. It becomes detectable between P8 and P12 and persists into adulthood. Synaptoporin is already detectable at P2. Synaptoporin in the same way as ZnT3 establishes as a strong marker for the mossy fiber tract into adulthood. Expression of the general excitatory synaptic marker VGLUT1 is already detectable at P2 at the different neuropilar regions of the hippocampus and maturates with development to the determined distribution.

D) Coronal sections from adult wild type mouse brains were double-immunostained for ZnT3 and Synaptoporin. Both antibodies yielded a nearly identical staining pattern of the mossy fiber tract demonstrating interchangeability of the two marker proteins for morphometrical measurements.


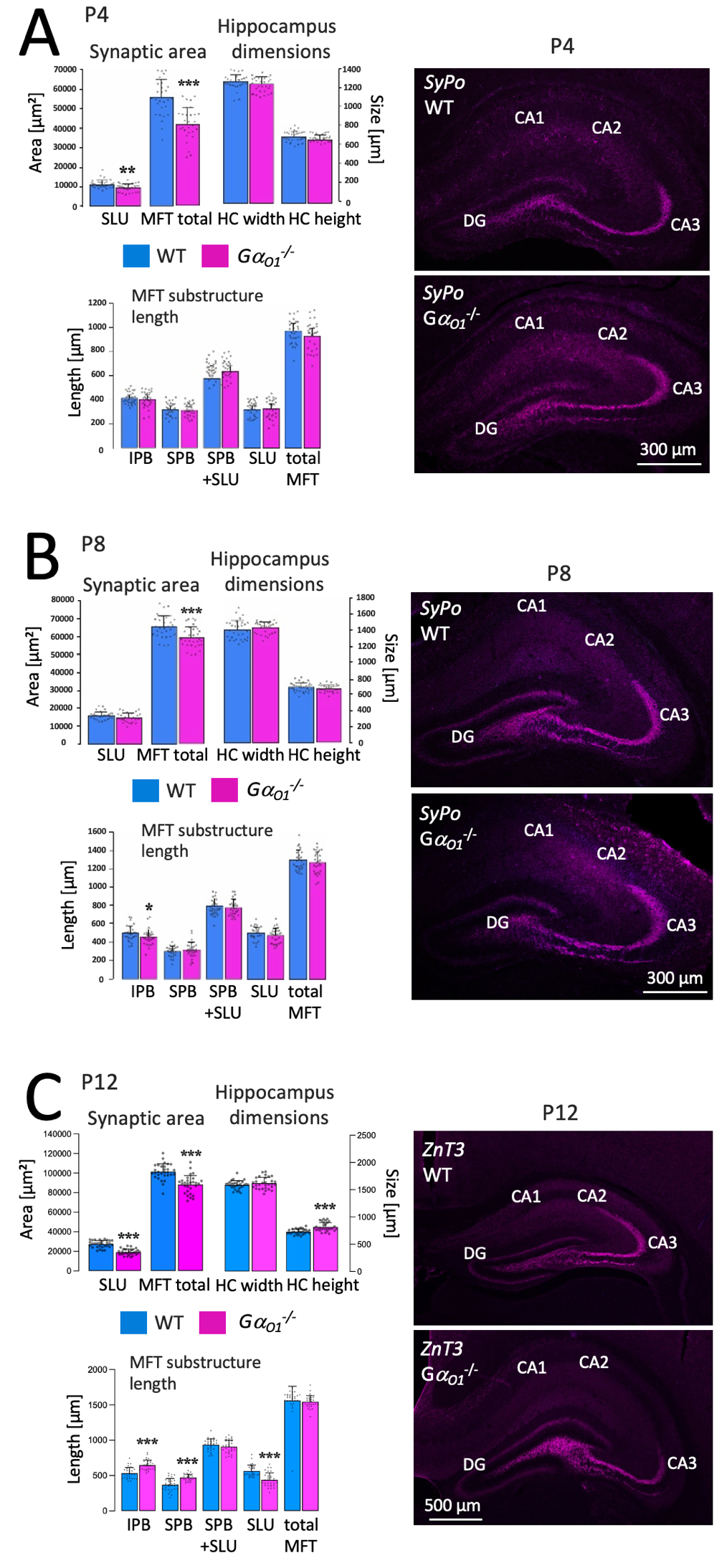


**Supplemental Figure 3**

**Development of differences in mossy fiber tract anatomy between wild type and Gα_o1_^-/-^** **mice**

Coronal brain sections from postnatal stages P4, P8 and P12 stained with Synaptoporin (SyPo) to visualize mossy fiber tract morphology. A) At P4, SLU and total synaptic area were significantly reduced in Gα_o1_^-/-^ mice, all other parameters remained unchanged, as judged by Synaptoporin stainings. B) At P8, total synaptic area and length of the infrapyramidal bundle (IPB) were the only parameters altered and showed a moderate reduction in Gα_o1_^-/-^ mice. C) At P12, total and SLU synaptic areas were significantly reduced together with a reduction in SLU length. In contrast, length of IPB, SBP and, very moderately, height of the hippocampus were increased in Gα_o1_^-/-^ mice. Bars show means ± SD from N = 3 pooled animals each WT and Gα_o1_^-/-^ per age, 30 mossy fiber tracts per genotype and age. *P≤0,05; **P≤0,01; ***P≤0,001


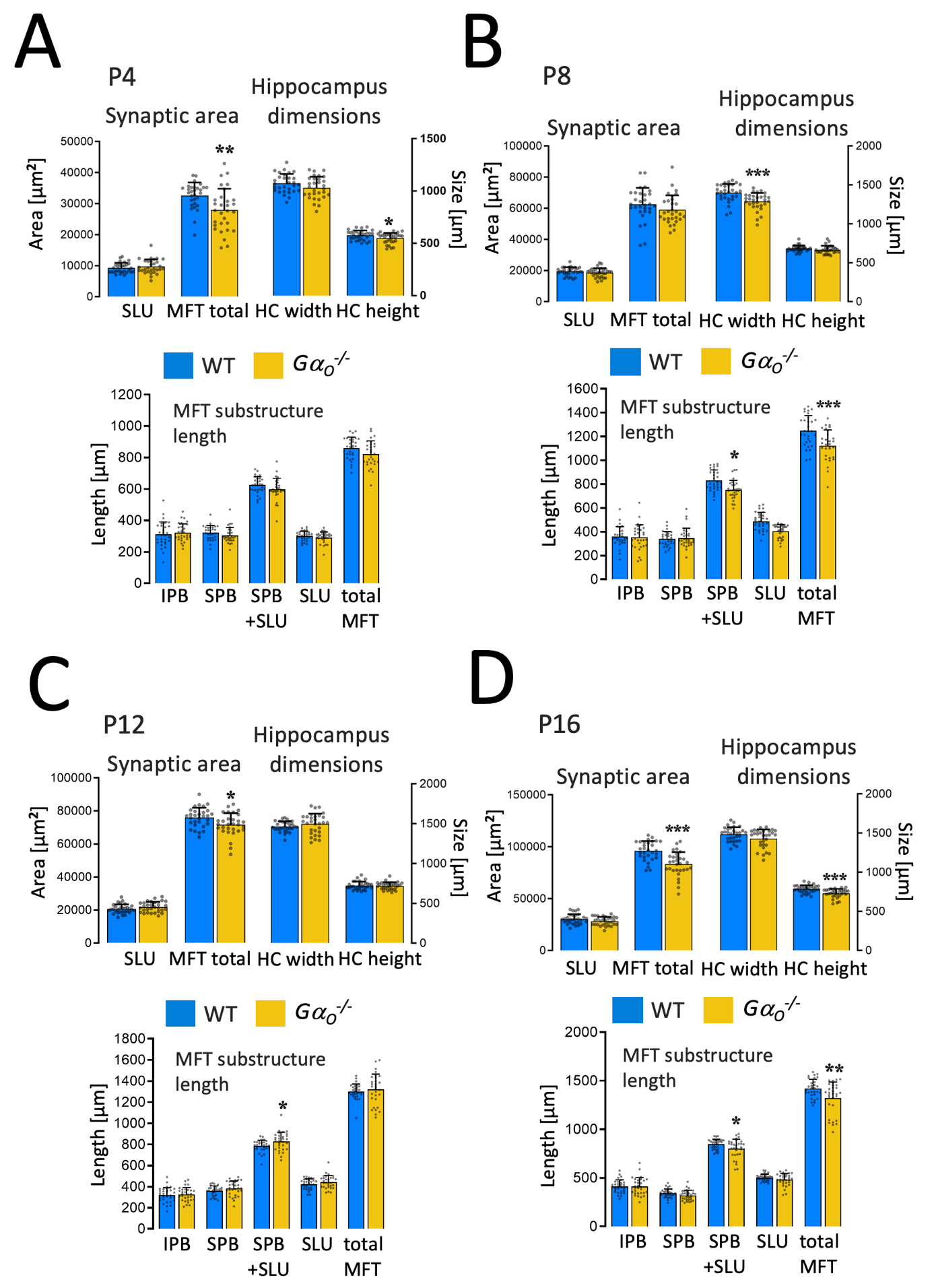


**Supplemental Figure 4**

**Development of the mossy fiber tract anatomy in wild type and Gα_o_^-/-^** **mice**

Measurements of mossy fiber tract parameters of wild type and Gα_o_^-/-^ mice from postnatal day 4 to P16. Analysis was based on Synaptoporin stainings. A) At P4, moderate differences in the size of the synaptic area of the total mossy fiber tract (MFT) were observed. All other MFT parameters analyzed remained unchanged. B) At P8, the combined length of the SPB and SLU as well as the total MFT length were moderately reduced in Gα_o_^-/-^ mice, all other parameters remained unchanged. C) At P12, the total synaptic area was slightly reduced in the knockout while the combined length of SPB and SLU was very slightly increased in Gα_o_^-/-^ mice. D) At P16, total MFT synaptic area remained significantly reduced. At the same time, combined SBP as well as the total MFT length were moderately reduced in Gα_o_^-/-^ mice.

Bars show means ± SD from N = 3 pooled animals each WT and Gα_o_^-/-^ per age, 30 mossy fiber tracts per genotype and age. *P≤0,05; **P≤0,01; ***P≤0,00
