# Supplementary material for: Gαo1 and Gαo1/Gαo2 deletion differentially affect hippocampal mossy fiber tract anatomy and neuronal morphogenesis
Source: J Neurochem. 2024 Oct 28;169(2):e16248. doi: 10.1111/jnc.16248 (PMC11808459; doi:10.1111/jnc.16248)
Supplement: Supplementary file 7 — Data S3. [file JNC-169-0-s007.pdf]

$G\alpha_{o1}$ and $G\alpha_{o1}$ / $G\alpha_{o2}$ deletion differentially affect hippocampal mossy fiber tract anatomy and neuronal morphogenesis

Markus Höltje[1]*, Anton Wolkowicz[1], Irene Brunk[1], Jens Baron[1], and Gudrun Ahnert-Hilger[2]

[1] Institut für Integrative Neuroanatomie, Charité-Universitätsmedizin Berlin, corporate member of Freie Universität Berlin and Humboldt-Universität zu Berlin
[2] Laboratory of Neurobiology, Max-Planck-Institute for Biophysical Chemistry and University of Göttingen, Germany

Supplemental data

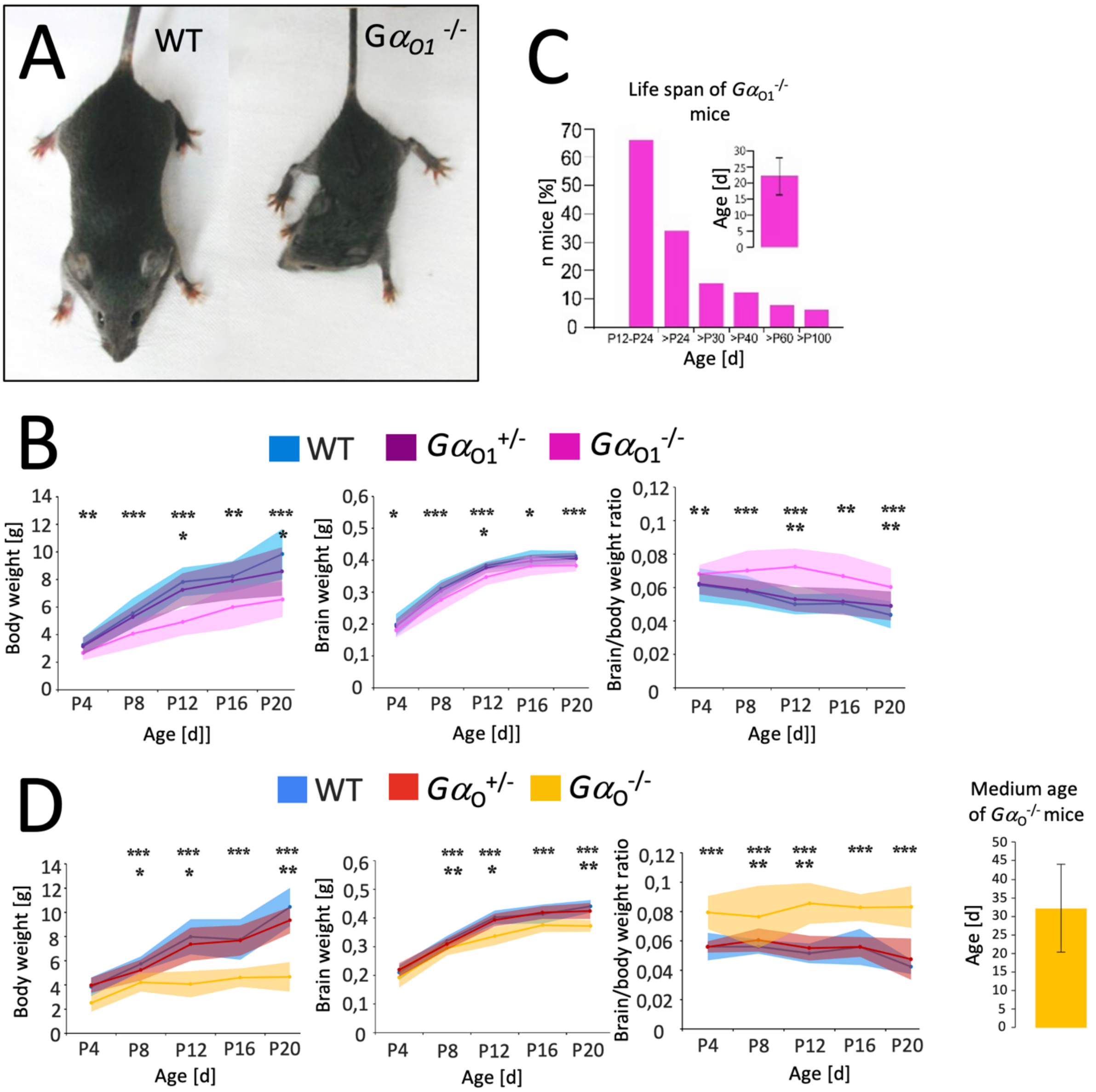

Supplemental Figure 1

Single $G\alpha_{o1}$ and double $G\alpha_{o1}$ / $G\alpha_{o2}$ knockout result in growth retardation and reduced live span

A) Phenotypical appearance of adult wild type and $G\alpha_{o1}^{-/-}$ mice. Homozygous $G\alpha_{o1}$ knockout mice exhibit a marked retardation in body size. B) Time course of postnatal body weight development in wild type, $G\alpha_{o1}^{+/-}$ and $G\alpha_{o1}^{-/-}$ mice (left panel). Determination of body weight was performed at postnatal (P) days P4, P8, P16 and P20. At all time points analyzed, homozygous mice were significantly lighter than wild type animals. Heterozygous mice showed significant alterations in body weight at P12 and P20. Corresponding postnatal development

of brain weight in wild type, $G\alpha_{o1}^{+/-}$ and $G\alpha_{o1}^{-/-}$ mice (middle panel). At all time points analyzed, brains of homozygous mice were moderately lighter than the ones of wild type and heterozygous animals. Heterozygous mice showed no significant alterations in brain weigh compared to the wild type except for P12. Brain / body weight rations during postnatal development (right panel). The strong reduction in body size of homozygous mice compared to the relatively small reduction in brain size is reflected by the given ratios. C) Survival rates (given in %) of $G\alpha_{o1}^{-/-}$ mice at indicated postnatal time periods. Less than 10% of the animals survive to ages older than 100 days. Median age was calculated to 22,2 days. Data in B) are given as means ± SD (SD shown as transparent areas); N= 16-55 animals per condition of either sex. *P≤0,05; **P≤0,01; ***P≤0,001 upper asterisks wt vs. homoz KO., lower asterisks wt vs. heteroz KO C) N=37 animals D) Time course of postnatal body weight development (left panel) in wild type, $G\alpha_{o}^{+/-}$ and $G\alpha_{o}^{-/-}$ mice. Determination of body weight was performed at postnatal days P4, P8, P12, P16 and P20. From P8 on, homozygous mice were significantly lighter than wild type and heterozygous animals. Heterozygous mice showed only slight alterations in body weight at individual time points. Corresponding postnatal development of brain weight (middle panel). Also from P8 on, brains of homozygous mice were moderately lighter than the ones of wild type and heterozygous animals. Heterozygous mice showed very slight reductions in brain weigh compared to the wild type at individual time points. Brain/ body weight rations during postnatal development (right panel). The strong reduction in body size of homozygous mice compared to the relatively small reduction in brain size is reflected by the given ratios. Data are given as means ± SD (SD shown as transparent areas); N= 14-123 animals per condition of either sex. Median age was calculated to 32,1 days. *P≤0,05; ***P≤0,01; ***P≤0,001* upper asterisks wt vs. homoz KO., lower asterisks wt vs. heteroz KO

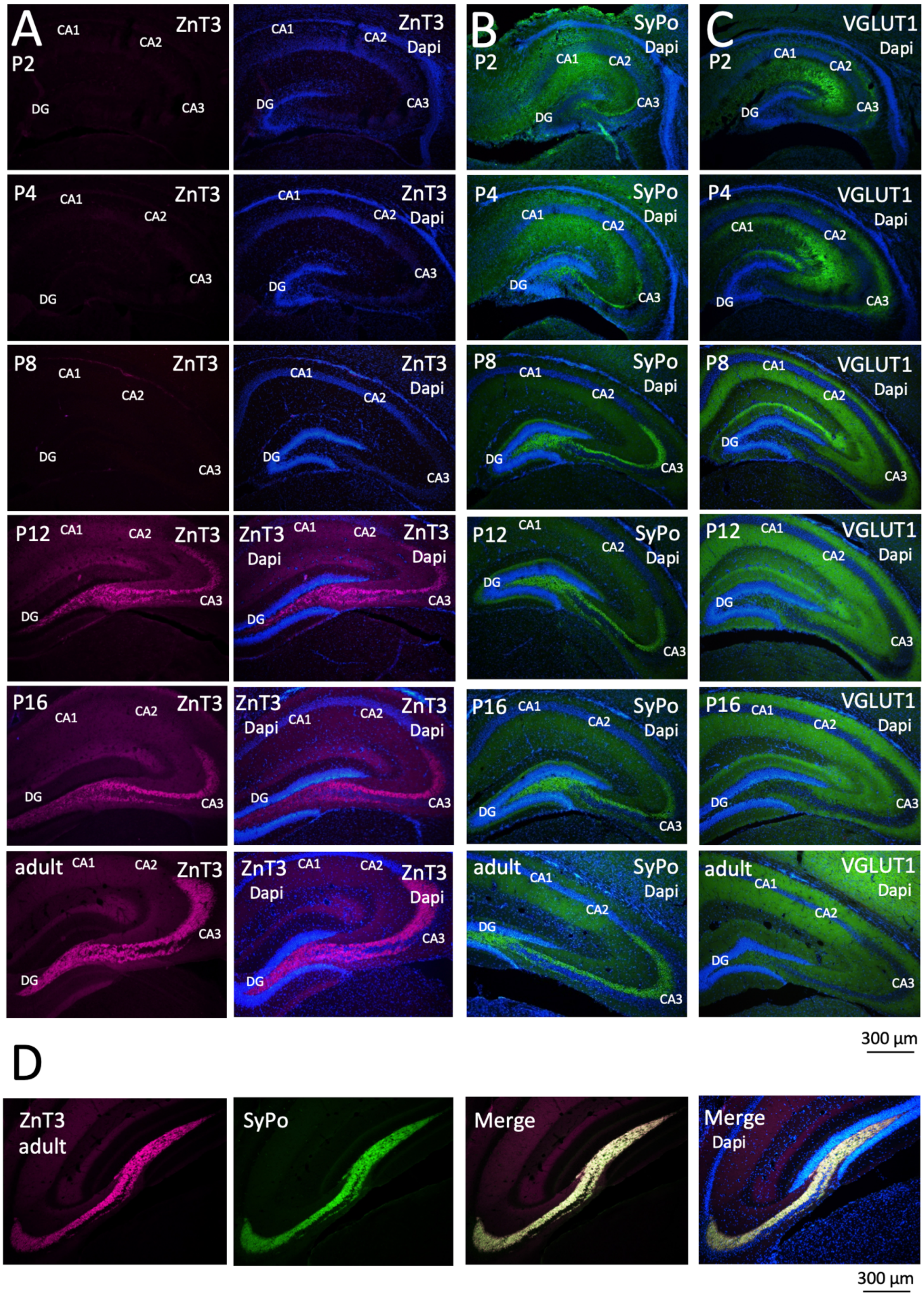
A
B
C
D
P2
P4
P8
P12
P16
adult
ZnT3
ZnT3 Dapi
SyPo Dapi
VGLUT1 Dapi
SyPo
Merge
Merge Dapi
ZnT3 adult
CA1
CA2
CA3
DG
300 µm
300 µm

Supplemental Figure 2

Postnatal development of mossy fiber tract marker proteins and the general excitatory synaptic marker VGLUT1

Coronal sections from postnatal days 2, 4, 8, 12, 16 and adult wild-type mouse brains were immunostained for either ZnT3 (A), Synaptoporin (SyPo, B), or vesicular glutamate transporter 1 (VGLUT1, C). Immunohistochemical analysis shows that expression of ZnT3 is largely restricted to the mossy fiber tract. It becomes detectable between P8 and P12 and persists into adulthood. Synaptoporin is already detectable at P2. Synaptoporin in the same way as ZnT3 establishes as a strong marker for the mossy fiber tract into adulthood. Expression of the general excitatory synaptic marker VGLUT1 is already detectable at P2 at the different neuropilar regions of the hippocampus and maturates with development to the determined distribution.

D) Coronal sections from adult wild type mouse brains were double-immunostained for ZnT3 and Synaptoporin. Both antibodies yielded a nearly identical staining pattern of the mossy fiber tract demonstrating interchangeability of the two marker proteins for morphometrical measurements.

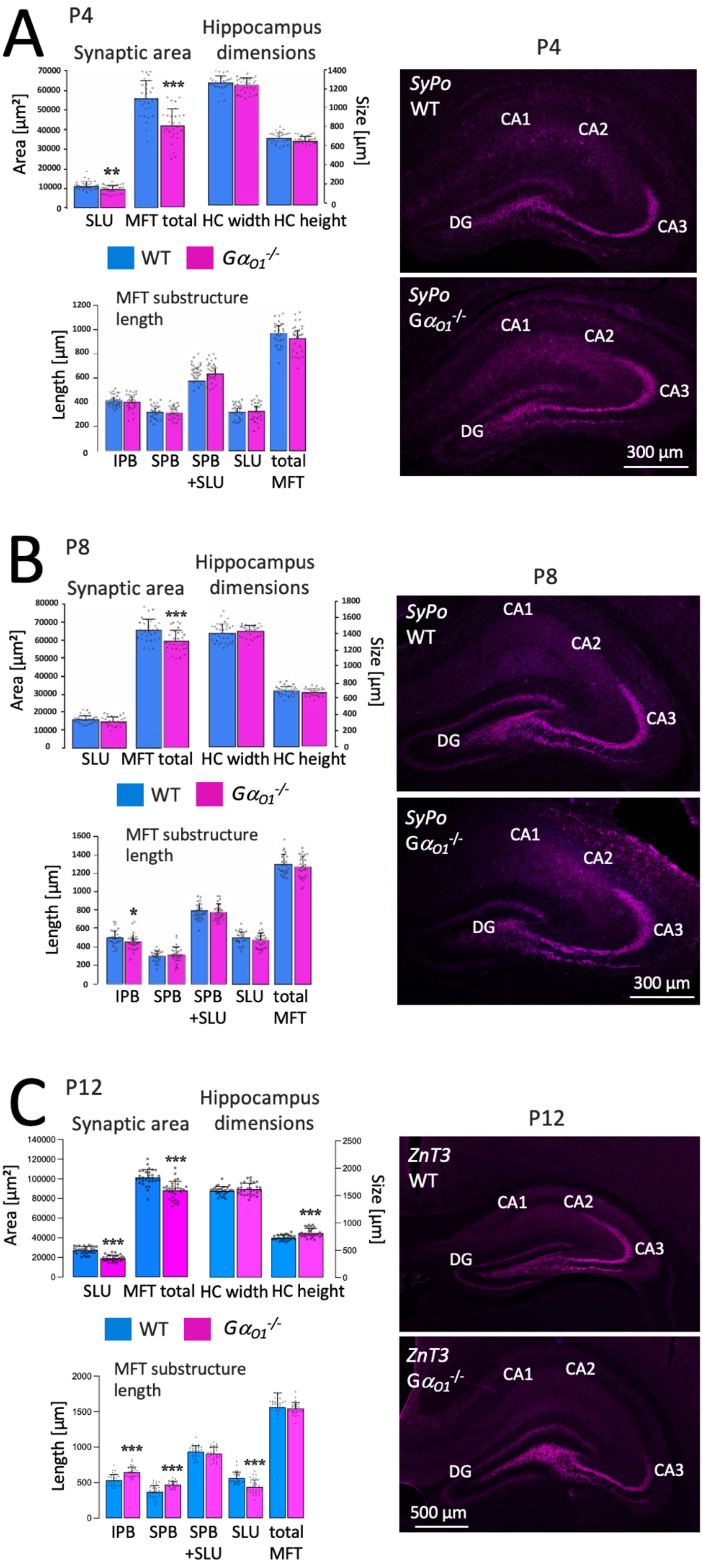
A
P4
Synaptic area
Hippocampus dimensions
Area [µm²]
Size [µm]
**
***
SLU
MFT total
HC width
HC height
WT
Gαo1-/-
MFT substructure length
Length [µm]
IPB
SPB
SPB +SLU
SLU
total MFT
P4
SyPo WT
CA1
CA2
DG
CA3
SyPo Gαo1-/-
300 µm
B
P8
*
SyPo WT
SyPo Gαo1-/-
C
P12
ZnT3 WT
ZnT3 Gαo1-/-
500 µm

Supplemental Figure 3

Development of differences in mossy fiber tract anatomy between wild type and $G\alpha_{o1}^{-/-}$ mice

Coronal brain sections from postnatal stages P4, P8 and P12 stained with Synaptoporin (SyPo) to visualize mossy fiber tract morphology. A) At P4, SLU and total synaptic area were significantly reduced in $G\alpha_{o1}^{-/-}$ mice, all other parameters remained unchanged, as judged by Synaptoporin stainings. B) At P8, total synaptic area and length of the infrapyramidal bundle (IPB) were the only parameters altered and showed a moderate reduction in $G\alpha_{o1}^{-/-}$ mice. C) At P12, total and SLU synaptic areas were significantly reduced together with a reduction in SLU length. In contrast, length of IPB, SBP and, very moderately, height of the hippocampus were increased in $G\alpha_{o1}^{-/-}$ mice. Bars show means ± SD from N = 3 pooled animals each WT and $G\alpha_{o1}^{-/-}$ per age, 30 mossy fiber tracts per genotype and age. *P≤0,05; **P≤0,01; ***P≤0,001

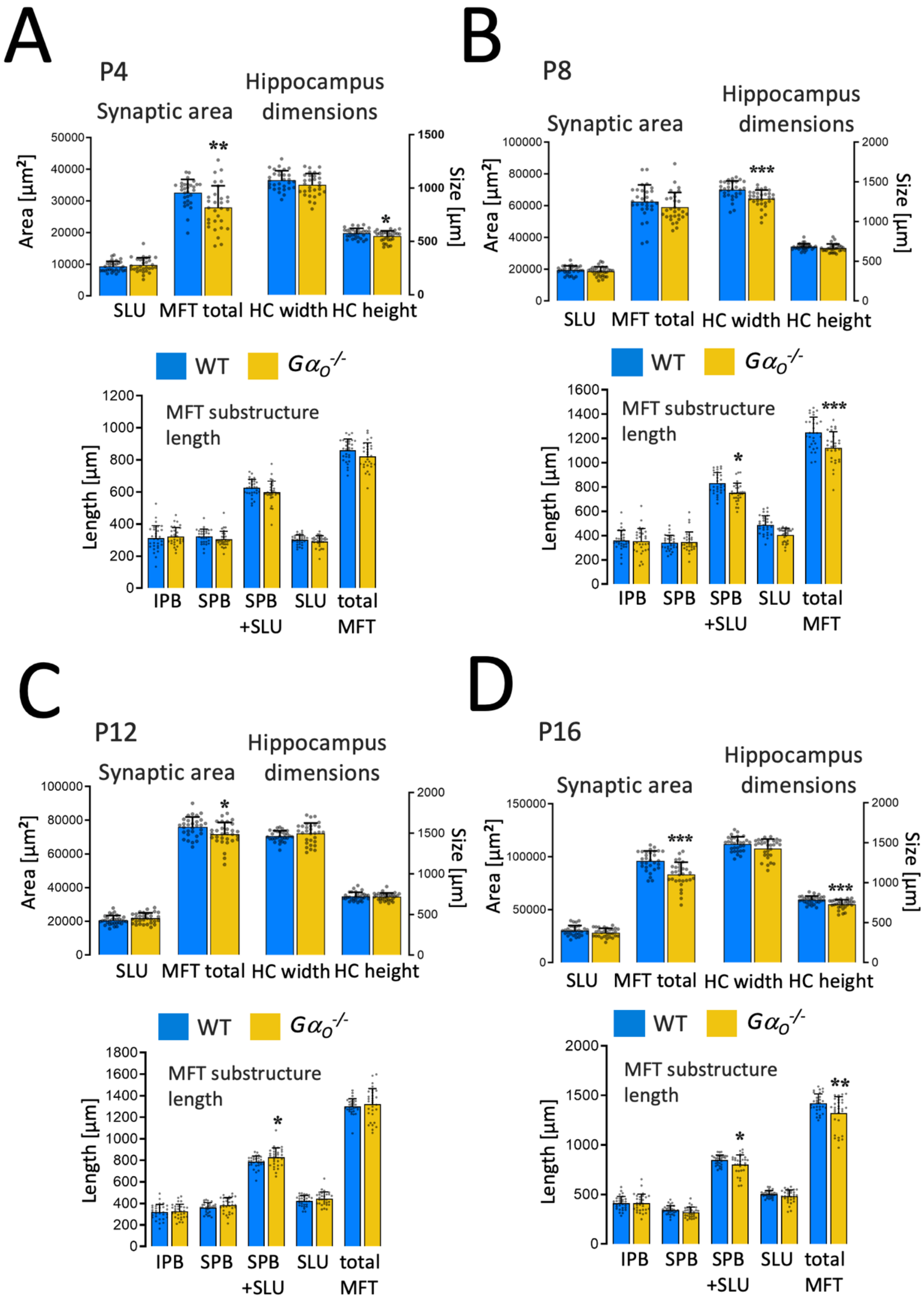

A
P4
Synaptic area
Hippocampus dimensions
Area [µm²]
Size [µm]
SLU
MFT total
HC width
HC height
WT
Gαo-/-
MFT substructure length
Length [µm]
IPB
SPB
SPB +SLU
SLU
total MFT
B
P8
C
P12
D
P16

Supplemental Figure 4

Development of the mossy fiber tract anatomy in wild type and $G\alpha_o^{-/-}$ mice

Measurements of mossy fiber tract parameters of wild type and $G\alpha_o^{-/-}$ mice from postnatal day 4 to P16. Analysis was based on Synaptoporin stainings. A) At P4, moderate differences in the size of the synaptic area of the total mossy fiber tract (MFT) were observed. All other MFT parameters analyzed remained unchanged. B) At P8, the combined length of the SPB and SLU as well as the total MFT length were moderately reduced in $G\alpha_o^{-/-}$ mice, all other parameters remained unchanged. C) At P12, the total synaptic area was slightly reduced in the knockout while the combined length of SPB and SLU was very slightly increased in $G\alpha_o^{-/-}$ mice. D) At P16, total MFT synaptic area remained significantly reduced. At the same time, combined SBP as well as the total MFT length were moderately reduced in $G\alpha_o^{-/-}$ mice.

Bars show means ± SD from N = 3 pooled animals each WT and $G\alpha_o^{-/-}$ per age, 30 mossy fiber tracts per genotype and age. $^*P \leq 0,05$; $^{**}P \leq 0,01$; $^{***}P \leq 0,00$
